# Discovery of Human Signaling Systems: Pairing Peptides to G Protein-Coupled Receptors

**DOI:** 10.1016/j.cell.2019.10.010

**Published:** 2019-10-31

**Authors:** Simon R. Foster, Alexander S. Hauser, Line Vedel, Ryan T. Strachan, Xi-Ping Huang, Ariana C. Gavin, Sushrut D. Shah, Ajay P. Nayak, Linda M. Haugaard-Kedström, Raymond B. Penn, Bryan L. Roth, Hans Bräuner-Osborne, David E. Gloriam

**Affiliations:** 1 Department of Drug Design and Pharmacology, University of Copenhagen, Universitetsparken 2, 2100 Copenhagen, Denmark; 2 Department of Pharmacology, University of North Carolina at Chapel Hill School of Medicine, Chapel Hill, NC 27599, USA; 3 Department of Pharmacology, School of Medicine, and the Division of Medicinal Chemistry and Chemical Biology, Eshelman School of Pharmacy, and the NIMH Psychoactive Drug Screening Program, University of North Carolina at Chapel Hill, Chapel Hill, NC 27599, USA; 4 Department of Medicine, Center for Translational Medicine and Division of Pulmonary, Allergy and Critical Care Medicine; Jane and Leonard Korman Respiratory Institute, Thomas Jefferson University, Philadelphia, PA 19107, USA

**Keywords:** GPCR, peptide ligand, endogenous ligand, deorphanization, orphan receptor, genomics, evolution, pharmacological screening, receptor internalization, machine learning

## Abstract

The peptidergic system is the most abundant network of ligand-receptor-mediated signaling in humans. However, the physiological roles remain elusive for numerous peptides and more than 100 G protein-coupled receptors (GPCRs). Here we report the pairing of cognate peptides and receptors. Integrating comparative genomics across 313 species and bioinformatics on all protein sequences and structures of human class A GPCRs, we identify universal characteristics that uncover additional potential peptidergic signaling systems. Using three orthogonal biochemical assays, we pair 17 proposed endogenous ligands with five orphan GPCRs that are associated with diseases, including genetic, neoplastic, nervous and reproductive system disorders. We also identify additional peptides for nine receptors with recognized ligands and pathophysiological roles. This integrated computational and multifaceted experimental approach expands the peptide-GPCR network and opens the way for studies to elucidate the roles of these signaling systems in human physiology and disease.

**Video Abstract:**

## Highlights

• Universal characteristics enabled prediction of peptide ligands and receptors
• Multifaceted screening enabled detection of pathway- and assay-dependent responses
• Peptide ligands discovered for BB_3_, GPR1, GPR15, GPR55, and GPR68
• Each signaling system is a link to human physiology and is associated with disease

## Graphical Abstract

**Figure:**
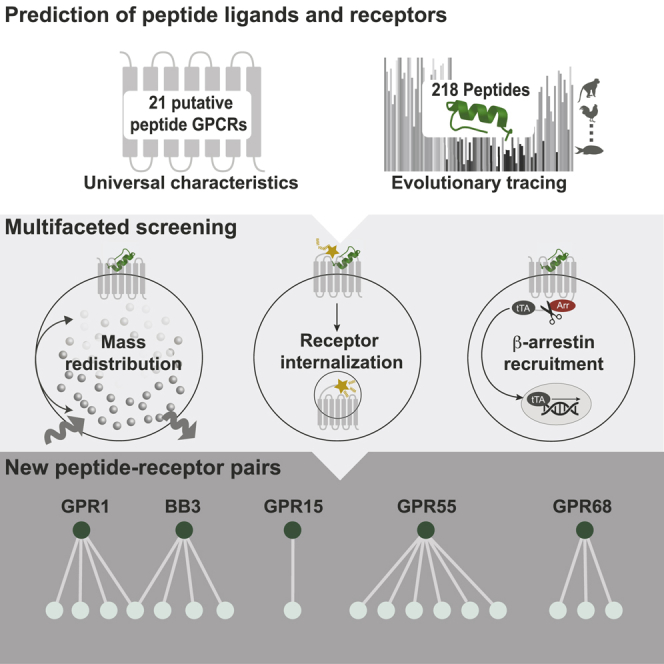

## Teaser

Features learned from comparative sequence and structural analyses enabled prediction of peptide ligands for orphan GPCRs that, when coupled with functional validation, expose physiologically relevant signaling systems.

## Introduction

Peptide hormones and neuropeptides are ubiquitous signaling molecules that predominantly stimulate cell surface receptors in numerous physiological processes. Over 85 endogenous peptide/protein-derived drugs target 51 proteins, half of which are G protein-coupled receptors (GPCRs) (Wishart et al., 2018). Moreover, such biological agents and peptide-activated GPCRs are gaining traction in current clinical trials (Hauser et al., 2017). However, despite their physiological importance and therapeutic potential, the cognate interactions for numerous peptides and over 100 GPCRs remain elusive; thus, they are referred to here as “orphan” receptors (Laschet et al., 2018) or, for simplicity, “oGPCRs.”

Deorphanization, i.e., unambiguous pairing of cognate ligands and receptors, has consistently transformed the understanding of human biology (Civelli et al., 2013), and illumination of understudied drug targets is a key objective of modern drug discovery (Oprea et al., 2018, Roth and Kroeze, 2015). However, deorphanization has been slow in recent years (https://www.guidetopharmacology.org/latestPairings.jsp). Furthermore, oGPCRs typically have uncharacterized signaling pathways (Roth et al., 2017), necessitating the use of promiscuous G proteins (Huang et al., 2015) and β-arrestin assays to report cellular responses (Kroeze et al., 2015, Southern et al., 2013). Because not all GPCRs couple efficiently to promiscuous/chimeric G proteins and/or may not robustly induce β-arrestin recruitment, these assays may miss many *bona fide* receptor-ligand interactions. Equally, the pluridimensional nature of GPCR signaling and the ability of some ligands to preferentially activate one signaling pathway at the expense of others (i.e., to bias their stimulus) requires the use of multiple complementary assays to effectively study oGPCRs.

Human peptide ligands, such as QRFP peptides, osteocalcin, and spexin, were discovered using bioinformatics approaches (Fukusumi et al., 2003, Mirabeau et al., 2007, Sonmez et al., 2009), which have the ability to interrogate complete genomes. However, bioinformatics approaches must account for conceptual challenges related to biological processes, including identification of signal peptides for secretion, alternative splicing of precursor genes, and enzymatic peptide cleavage (Ozawa et al., 2010). Furthermore, post-translational modifications and protein folding are generally not covered by computational methods, although some sequence motif-based modifications can be predicted, such as the introduction of C-terminal amidation and disulfide bridges. Mass spectrometry-based techniques have been used to discover endogenous ligands in the mouse (Fricker, 2010) and the human precursor proSAAS (Fricker et al., 2000), which contains bioactive peptides involved in circadian rhythms (Hatcher et al., 2008). However, mass spectrometry can be limited in terms of detection of low-abundance peptides in complex samples.

**Figure S1:**
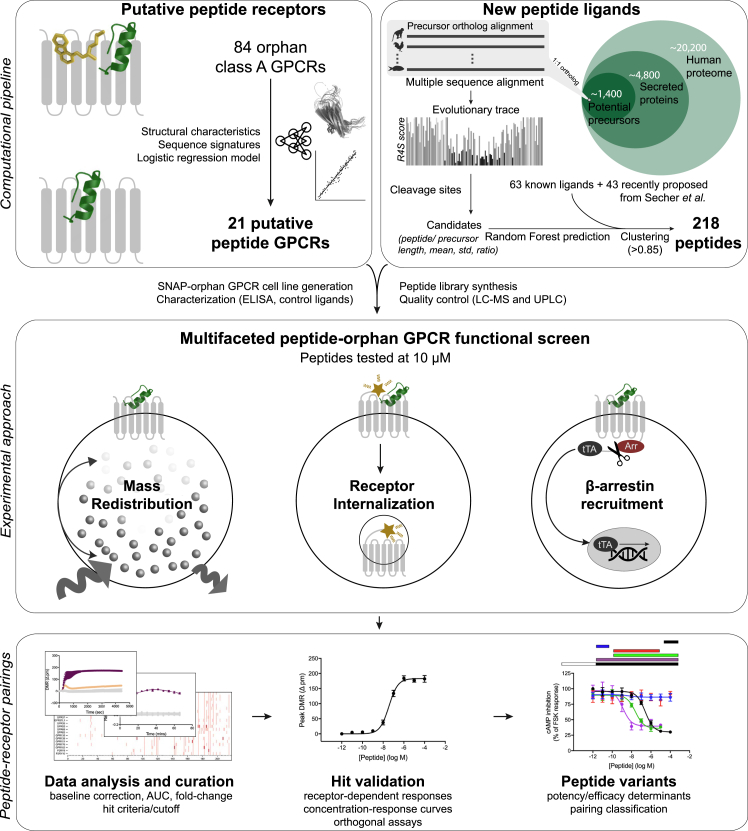
Peptide-Receptor Pairing Approach, Related to Figures 2, 3, 4, and 5 21 orphan receptor targets were selected based on shared characteristics of known peptide-activated GPCRs. A library of 218 peptides was generated using a proteome-wide machine-learning approach. Peptides were screened using three complementary functional assays. Putative peptide-oGPCRs pairings were validated using additional assays. Predicted cleavage variants of discovered peptide agonists were tested to gain insights into determinants of peptide potency.

Here we provide an integrated computational and experimental approach for peptide-oGPCR pairing (Figure S1). We initially utilized comparative sequence and structural analyses to gain biological insights into the human peptide-receptor signaling landscape and leveraged these features to mine candidate peptide ligands in the human genome. We then identified interactions via multiple orthogonal assay platforms to independently screen class A GPCRs against key signal transduction events. Ultimately, we discovered potential endogenous peptide ligands for five oGPCRs as well as secondary ligands for a number of known peptide receptors.

## Results

### Cognate Peptide Ligands and Receptors Co-evolved to Form the Largest Signal Transduction System in Humans

**Figure 1:**
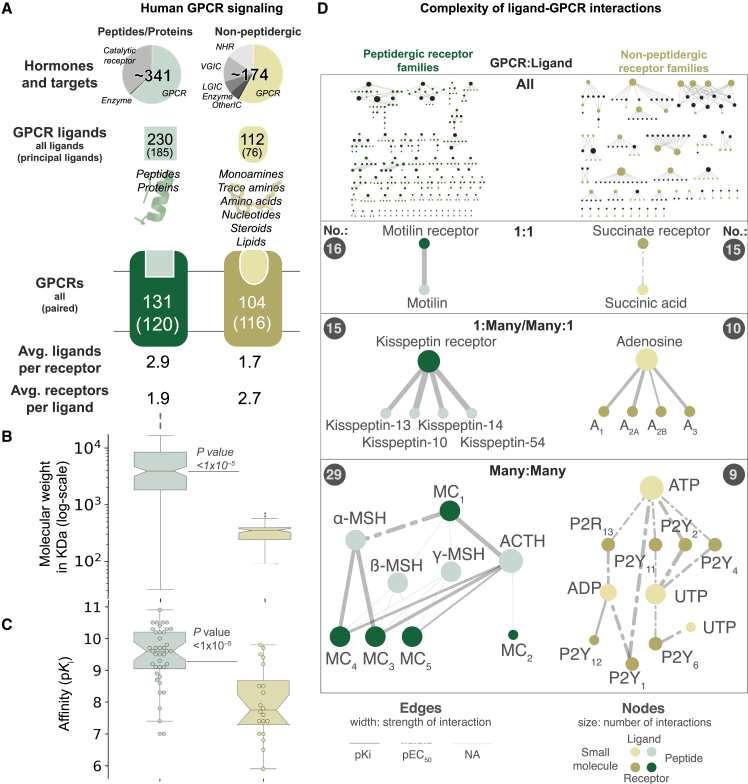
The Human G Protein-Coupled Receptor-Ligand System (A) GPCRs represent the predominant targets for endogenous ligands. Peptides are more numerous, larger and bind with higher affinity than non-peptide ligands. From the top: (1) distinct endogenous ligands by target family; (2) endogenous GPCR ligands, of which “principal” ligands are considered most physiologically relevant; (3) peptide and small-molecule binding receptors, of which “paired” ones have a known principal endogenous ligand; and (4) ligands per receptor and vice versa (averages). (B and C) Ligand molecular weight distribution (B) and cognate receptor affinity (C) (boxplots show a median and interquartile range of 1.5; Wilcoxon rank-sum test, p < 1 × 10^−5^). Data are from the Guide to Pharmacology database (Harding et al., 2018). (D) GPCR-ligand systems vary in complexity from 1:1 to many:many (gray circles show numbers of each system; data are shown in Table S1). See also Figure S2.

**Figure S2:**
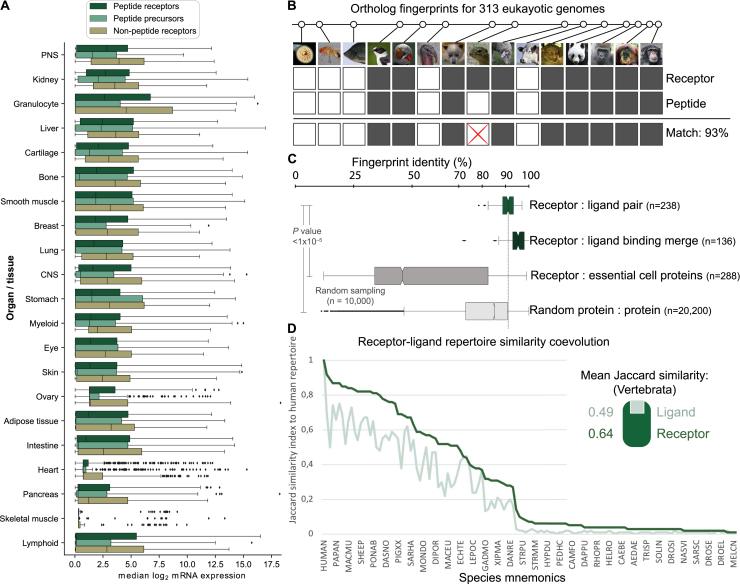
The Peptidergic Signaling System Has Been Shaped by Co-evolution of Ligands and Their Receptor Targets, Related to Figure 1 (A) Receptors and their ligands are ubiquitously expressed in human organs and tissues. The data is ordered by mean level of peptide receptor expression. Peptide receptors consistently had lower median expression levels than non-peptide GPCRs. Similar peptide receptor-ligand expression levels were observed for liver and smooth muscle, whereas granulocytes and lung tissue had higher expression of receptors than ligand precursors (Wilcoxon rank sum test; *P* values: < 1x10^−5^ (granulocytes) and 0.02 (lung)). (B) Evolutionary fingerprints indicated conservation (gray) or absence (white) of receptor and peptide precursor gene orthologs in 313 species (representatives shown). The fingerprint identity (%) reflects the evolutionary relationship of peptide-receptor pairs. Photos from Ensembl genome database project. (C) The average percentage identity of evolutionary receptor-ligand pairs for all endogenous receptor-ligand pairs is increased when fingerprints of ligands for the same receptor are merged and is greater than for a random protein pair (permutation tests by performing 10,000 randomizations, Wilcoxon rank sum test *P* value < 1x10^−5^). (D) Jaccard index similarity of human peptide receptor and ligand precursor repertoires (n = 131 and 130, respectively) to selected species ordered by evolutionary distance. Data in Tables S1 and S2.

Initially, we explored the current knowledge regarding endogenous ligands and receptor systems by evaluating 341 peptide/protein (encoded by 160 genes) and 174 non-peptide ligands (Harding et al., 2018). Both ligand classes mediate physiological functions predominantly through GPCRs (67% and 64%, respectively; Figure 1A; Table S1). The entire network of known interactions between GPCRs and cognate ligands spans 348 reported interactions between 120 receptors and 185 peptides. These interactions range from simple receptor-ligand systems with a one-to-one relationship to complex many-to-many systems (Figure 1D). For instance, the peptide hormone motilin signals through a single receptor, whereas the melanocortin and purinergic P2Y receptors are activated by multiple peptides and nucleotides, respectively. On average, each receptor is activated by 2.9 peptide or 1.7 non-peptidergic ligands. Peptides are larger (average molecular weight, 7.7 kDa versus 0.4 kDa) and interact with their targets with higher affinity (average p*K*_i_: 9.4 versus 8.0) and potency (average pEC/IC_50_: 9.0 versus 6.9) than non-peptides (Figures 1B and 1C; Table S1). The mRNA abundance is generally lower for peptide than non-peptide GPCRs (Figure S2A), although peptide ligand precursors and both types of receptors are expressed in all organs. Taken together, our most common and potent type of signaling molecules evolved through genetic encoding.

Next, we sought to investigate how the success of peptide ligands was shaped together with their cognate receptors (Mirabeau and Joly, 2013) by analyzing 23,606,407 peptide-GPCR relationships across 313 eukaryotic genomes. When considering the minimal signaling system of one peptide and receptor, we found that 39 of 42 (93%) of the known human families arose during vertebrate evolution (Table S2), consistent with two early genome duplications (Holland et al., 1994). Notably, among all receptor families in all species, few have only the precursor (4%) or GPCR (15%) gene, indicating nearly universal coevolution. Moreover, by generating evolutionary fingerprints of conserved or lost gene orthologs (Figure S2B), we observed significantly higher coevolution of cognate ligand-receptor than random protein-protein pairs (average identities of 91% and 56%, respectively). This coevolution is higher (average identities of 95% and 89%, respectively) when merging the fingerprints of peptide precursors, but not of GPCRs, within the same receptor family (Figure S2C). These results suggest higher evolutionary pressure to conserve the distinct physiological function of receptors than ligands. Similarly, we found that the human receptor repertoire is more conserved than peptide ligands (average J = 0.64 versus 0.49; Figure S2D). Thus, cognate peptides and GPCRs have coevolved, and ligands have been more adaptive than receptors in shaping new signaling systems.

### Precursors, Peptides, and Receptors Possess Universal Evolutionary, Sequence, and Structural Characteristics

**Figure 2:**
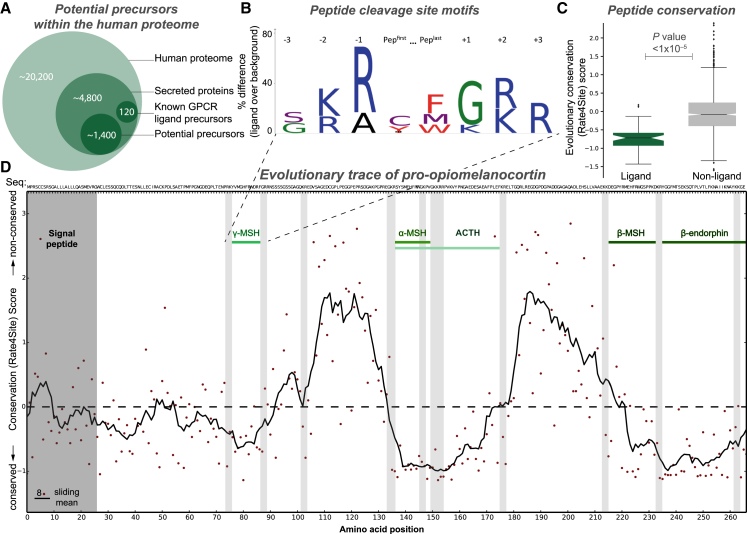
Universal Precursor Processing and Peptide Ligand Gene Conservation Hotspots (A) Potential precursors can be mined from the human proteome based on the presence of secretion signal peptides and an unknown or ligand-precursor-like function (Table S4). (B) The vast majority of GPCR peptide ligands are cleaved from precursors at specific dibasic sites. (C) GPCR peptide ligands are more evolutionarily conserved than random sequences of similar length (up to 45 residues) (Wilcoxon rank-sum test, p < 1 × 10^−5^). (D) Human peptide ligands can be deduced from precursor cleavage sites and conservation hotspots. The example depicts the pro-opiomelanocortin precursor containing endogenous ligands for melanocortin (α-MSH, β-MSH, γ-MSH, and ACTH) and opioid (β-endorphin) receptors.

We next explored whether comparative genomics and biological processing paradigms could be predictively used to identify peptide precursors and peptides. We found that 99% of all peptide ligand precursors contain an N-terminal signal peptide (Figure 2A), a requirement for extracellular secretion (Blobel and Dobberstein, 1975). Proteins are enzymatically cleaved at specific sites (Ozawa et al., 2010), and we found that 80%/66% of the 184 human peptide ligand N/C termini are flanked by a dibasic motif (with a conserved glycine at C termini; Figure 2B). In addition, evolutionary trace analysis showed that known peptides make up the most conserved segments of precursor sequences (Wilcoxon rank-sum test, p < 1 × 10^−5^; Figure 2C). Strikingly, the peptide ligand subsequences can be recognized precisely within their precursors as highly conserved segments flanked by consensus cleavage sites, the signal peptide, or the C terminus (Figure 2D).

**Figure 3:**
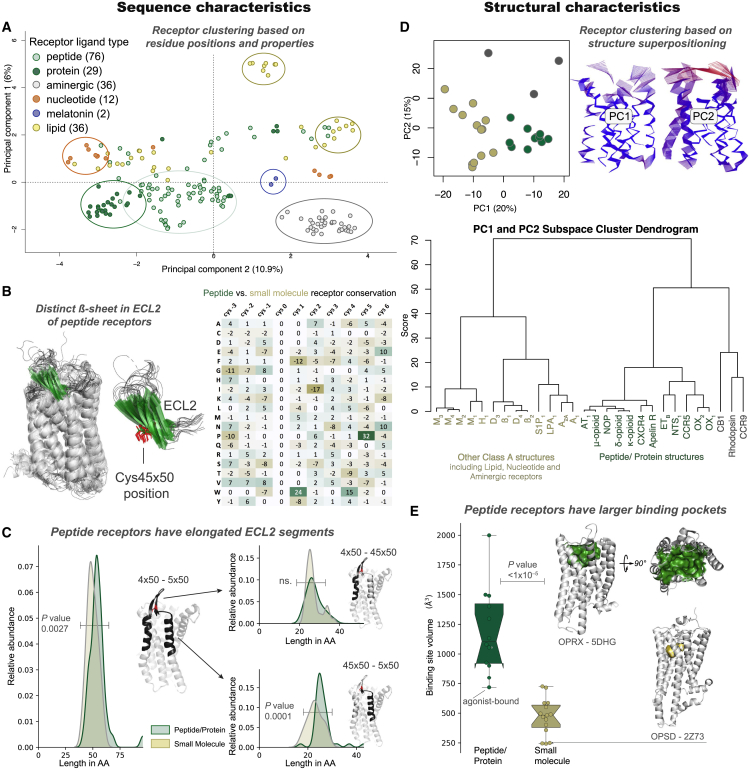
Peptide Receptors Share Distinct Sequence and Structural Characteristics (A) The majority of class A GPCRs cluster by endogenous ligand type based on ligand-interacting residue analysis with multi-dimensional scaling. (B) Peptide receptors with a structure (n = 21, left) share a characteristic β sheet (green) substructure (left) and sequence (right) in extracellular loop 2 (ECL2), which includes a conserved cysteine, Cys^45×50^ (red, center). (C) A long ECL2 segment (>20 residues) after Cys^45×50^ is an overrepresented feature of peptide/protein receptors (Wilcoxon rank-sum test, p < 1 × 10^−5^). (D) Principal-component analysis of receptor structures in a 2D plot (top left) and dendrogram (bottom) demonstrate separation of peptide (green), non-peptide receptors (beige), and outliers (gray). Differences are predominantly found in the extracellularly facing ligand-binding domain, as shown by residue displacements from the mean (right). (E) Ligand-binding pocket volumes are larger in peptide than non-peptide class A receptors. See also Table S3 for related 3D PCA and ECL2 motif data.

We then investigated the characteristic features of peptide receptors. By multi-dimensional scaling of residue properties across all aligned GPCR positions, we found that the vast majority of class A GPCRs cluster by ligand type and that protein and peptide receptors exhibit separate clusters (Figure 3A). GPCR structure analysis revealed a characteristic β sheet in the second extracellular loop with a distinct sequence conservation pattern (Figure 3B) and a long (>20 amino acid) distal segment of this loop that is twice as common in peptide receptors compared with non-peptide receptors (Figure 3C). Furthermore, principal-component analysis of GPCR structures revealed a clear separation of the peptide/protein receptors. Displacement trajectories of the two most significant principal components showed that the residue positions with the largest deviation from the average inactive structure are located within the extracellular portions of transmembrane helices 1–5 and the first two extracellular loops (Figure 3D). Moreover, these structures of peptide/protein receptors have nearly three times larger binding cavities than non-peptide GPCRs (mean volume, 1,226 Å^3^ and 469 Å^3^, respectively; Figure 3E). Taken together, these analyses reveal several sequence and structural characteristics, of which the β sheet, a long ECL2, and a large binding cavity can directly facilitate the binding of large proteins/peptides.

### Universal Characteristics Reveal Plausible Additional Human Peptides and Receptors

We sought to investigate whether it was possible to mine potential peptide ligands from the entire human proteome. First we identified putative precursors by sequentially filtering for proteins annotated in Swiss-Prot as secreted or with a signal peptide (∼4,800) and those with unknown or precursor-compatible functional annotations (∼1,400) (Figure 2A). This yielded 1,227 “peptide cleavage variants,” representing candidate ligands that span the precursor signal peptide and C terminus or intermediate consensus cleavage sites. We selected representative cleavage variants for pharmacological screening using a 5-fold cross-validated random forest classifier based on length and several evolutionary conservation scores of known peptides and their precursors. This analysis resulted in 120 peptide sequences representing the most plausible GPCR ligands. These were combined with 43 recently proposed unpaired rat peptides (Secher et al., 2016) to give a total of 163 putative peptide ligands, 112 of which came from precursors that have not been associated previously with GPCR activity. The final library containing 218 peptides was subsequently synthesized, including 55 known class A GPCR ligands (Table S5). To account for post-translational modification, we incorporated disulfide bridges and C-terminal amidation in 26 and 77 peptides, respectively. This synthetic peptide library contained the most GPCR ligand-like peptide cleavage variant, in effect “lead ligands” for primary screening.

**Figure S3:**
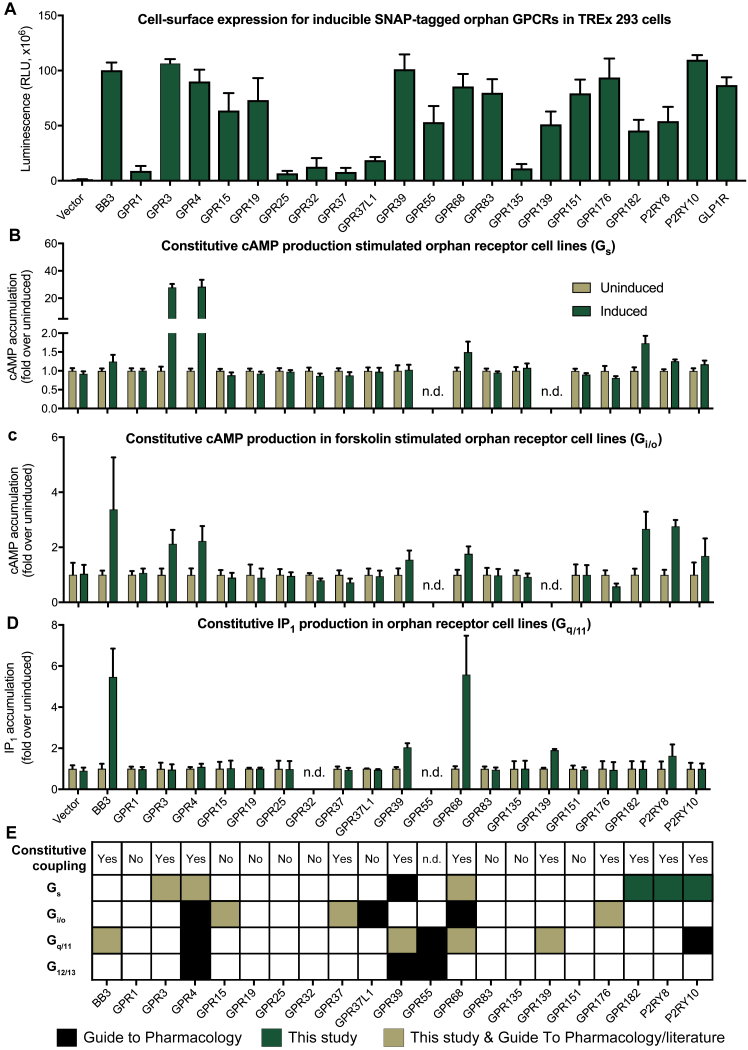
Receptor Expression and G Protein Coupling in Orphan GPCR Cell Lines, Related to Figure 4 (A) Cell-surface expression of induced oGPCRs measured by ELISA. Data shown as mean ± SEM from n = 3-7 independent experiments performed in triplicate. (B-D) Constitutive cAMP and IP_1_ production (i.e., in the absence of ligand) upon orphan receptor induction provide insights into G protein-coupling. Data shown as mean ± SEM from n = 3-5 independent experiments, except for BRS3/BB_3_ (n = 2 in (B) and (C)), GPR32 (n = 2 in (B)) and GPR3 (n = 2 in (C)). (E) For three receptors, we discovered constitutive G protein-coupling unreported in Guide to Pharmacology or literature (Doi et al., 2016, Harding et al., 2018, Inoue et al., 2012, Martin et al., 2015, Muppidi et al., 2014, Pera et al., 2018, Suply et al., 2017), and for GPR15 robust G_i/o_ signaling in cAMP accumulation assays.

In parallel, the conserved characteristics allowed us to predict peptide-activated receptors, of which we selected 21 class A GPCRs with rodent ortholog diverse disease associations (Table S5). Our cell lines for 15 (71%) oGPCRs displayed robust doxycycline-induced cell surface expression, and 12 (57%) promoted constitutive G protein signaling, including couplings for three receptors not reported previously (Figure S3). Furthermore, 10 oGPCR cell lines were validated with commercially available compounds (Table S5).

### A Multifaceted Screening Strategy Captures Pathway- and Assay-Dependent Receptor Responses

GPCRs can couple to multiple signaling pathways, with the combined signals constituting an overall response (Kenakin, 2017b). The measured response can appear different depending on the signal pathway, cell type, and time course investigated. Ligands can also intrinsically favor given receptor conformations that preferentially activate specific pathways (Kenakin, 2017b). These phenomena of assay-dependent observational bias and ligand-mediated signal bias are especially problematic for orphan and understudied receptors with poorly characterized signaling pathways (Huang et al., 2015, Roth et al., 2017).

For these reasons, we screened our 218 peptides and 21 predicted peptide receptors in three complementary orthogonal assay platforms to cover multiple aspects of GPCR activation. The dynamic mass redistribution assay is ideal for investigating oGPCR activation because it captures most (including all G protein) signaling pathways (Fang et al., 2008, Schröder et al., 2011). The second assay measures pathway-independent receptor internalization from the surface to the inside of cells (Foster and Bräuner-Osborne, 2018). These assays are both time resolved and provide valuable insights into receptor signaling kinetics. The third, the β-arrestin recruitment assay, is a highly amplified reporter gene-based readout of GPCR signaling (Kroeze et al., 2015) that increases the sensitivity of detecting positive pairings and allowed us to rapidly screen an additional 46 orphan/understudied and 27 known peptide-activated class A GPCRs (Table S6). Collectively, these three assays provided an ideal platform to detect peptide ligands for undercharacterized GPCRs and enabled physical interrogation of 21,446 potential peptide-receptor interactions.

**Figure 4:**
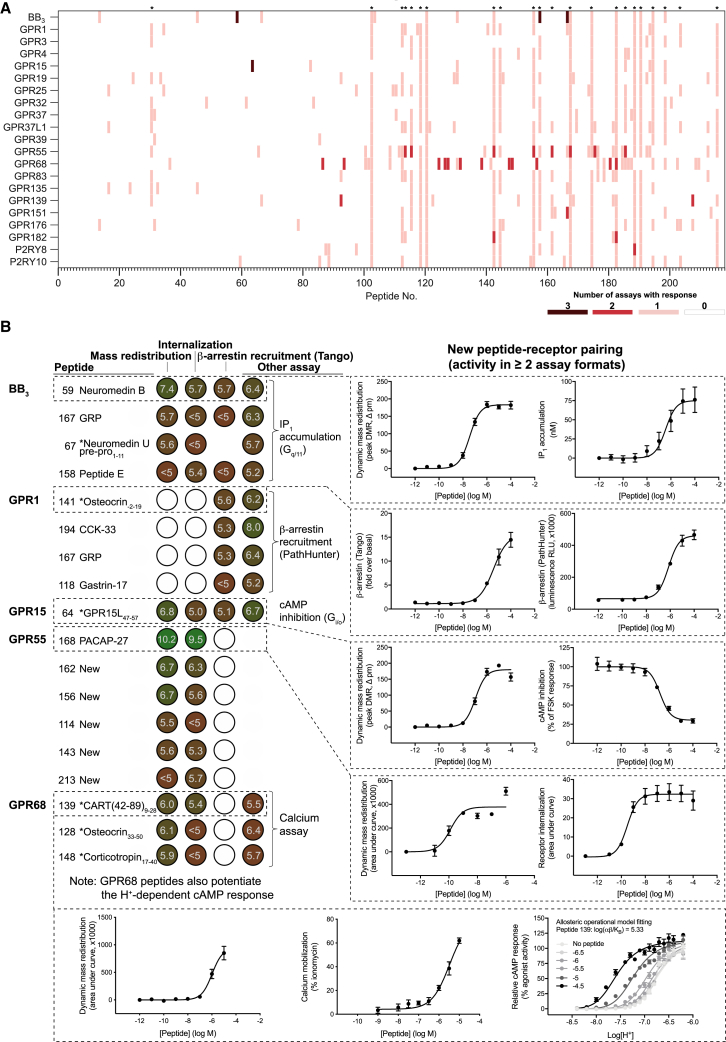
General versus Assay-Specific Responses and Novel Peptide-Receptor Pairings (A) The multifaceted screen of 218 peptides identified a variety of multiple and single-assay responses, including hits for all 21 predicted peptide receptors. Mass redistribution data revealed repeat hitters (denoted with asterisks) that reflect peptide-dependent responses from endogenous targets. Screening results for additional class A orphan and peptide GPCRs are provided in Table S6. (B) Pairing of 17 peptides with five orphan receptors. Colored circles show pEC_50_ values and concentration-response curves the most potent ligand for each receptor. Other assays used were G_q/11_ (IP_1_), G_s_ and G_i/o_ (cAMP), and β-arrestin recruitment (PathHunter). An asterisk indicates a new cleavage variant of a known GPCR peptide ligand with the amino acid range of the cleaved peptide shown in subscript; empty circles indicate inactivity. All data represent mean ± SEM for 3–4 independent experiments, each performed in triplicate. Table S7 provides all related data and data for indicative pairings (tested in a single assay) for GPR17, GPR161, GPR176, GPR183, and MAS1.

We confirmed the activity of known agonists for 21 (78%) recognized peptide receptors using the β-arrestin recruitment assay. Our multifaceted screening identified peptide-mediated responses for all 21 predicted orphan peptide receptors (Figure 4A; Table S6). These results validate our computational peptide library design and provide good experimental coverage of receptor signaling. The three screening platforms demonstrated large variation in peptide pairings/GPCR targets (receptor internalization, 24/6; β-arrestin recruitment, 57/21; and mass redistribution, 75/18; Table S6). We identified peptides that robustly activated bombesin receptor 3 (BB_3_), GPR1, GPR15, GPR55, and GPR68 in multiple primary assays (Figure 4A; Table S6). Notably, all GPR55 and GPR68 peptides were inactive in β-arrestin recruitment, and, conversely, GPR1 pairings were only observed in this assay, indicating potential ligand-mediated G protein and β-arrestin signal pathway bias, respectively. For the additional receptors only screened in the β-arrestin recruitment assay, we observed hits for 33 (72%) oGPCRs and nine known peptide receptors (Table S6). These findings underscore the importance of broadly covering receptor signaling using a multifaceted primary screening approach and additional functional assays for hit validation (Huang et al., 2015, Kroeze et al., 2015).

### Discovery of Peptide-Receptor Pairs Expand the Human Signaling System

**Figure S4:**
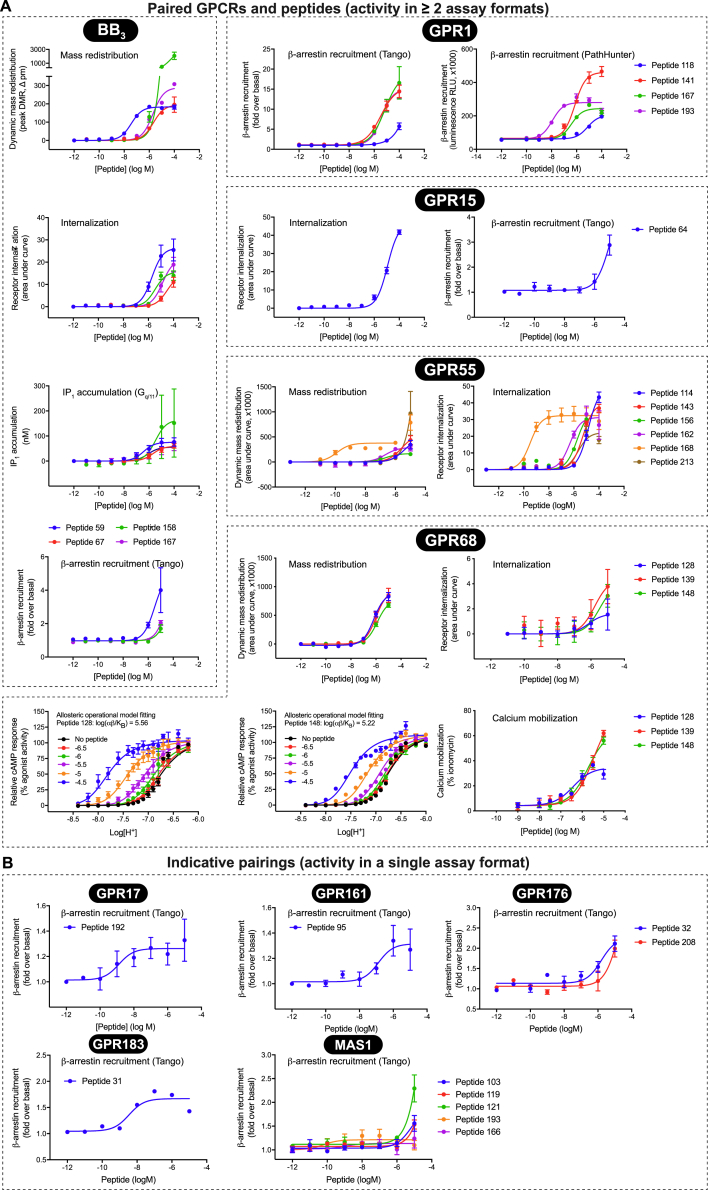
Peptide-GPCR Pairings across Multiple Assay Formats, Related to Figures 4 and 5 (A) Concentration-response measurements of BB_3_, GPR1, GPR15, GPR55 and GPR68 peptide ligands with activity in at least two assays. Assays used were ligand-dependent dynamic mass redistribution, receptor internalization (TR-FRET), G_q/11_ (IP_1_), G_s_ (GloSensor cAMP) and β-arrestin recruitment (Tango and PathHunter). (B) Indicative pairings from a single assay (Tango) for additional receptor targets. All data represent mean ± SEM for n = 3-4 independent experiments performed in triplicate, except for BB_3_/peptide 158 in IP_1_ accumulation (n = 2) and GPR55/peptide 156 (n = 1). Pharmacological parameters are provided in Table S7.

**Figure 5:**
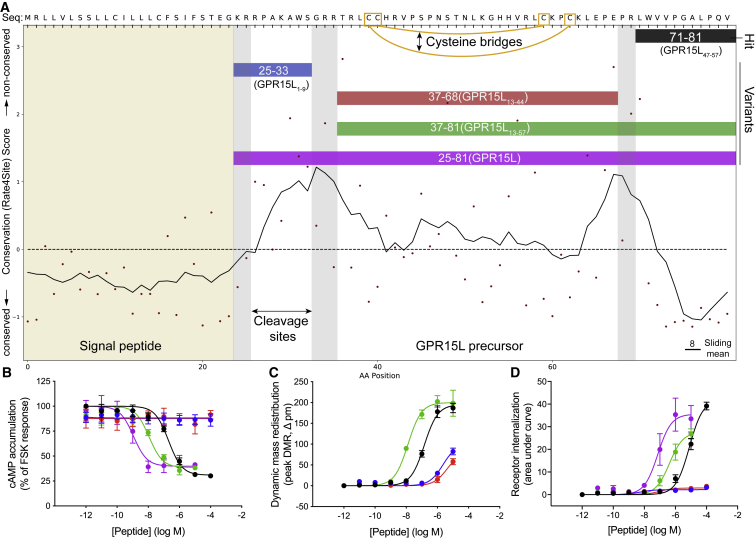
Potential Peptide Cleavage Variants Elicit Increased GPR15 Signaling Responses (A) Evolutionary trace and cleavage site (gray bars) analysis of the *GPR15L* gene-encoded precursor presents potential alternative peptide cleavage variants. (B–D) GPR15-mediated responses for *GPR15L* cleavage variants in (B) cAMP inhibition, (C) mass redistribution, and (D) receptor internalization assays. The most potent peptide is the longest, 57-residue form the recently named “GPR15L” (Suply et al., 2017) (excluded in C because of assay interference). Data represent mean ± SEM for 3–4 independent experiments performed in triplicate. Related pharmacological data for GPR15 as well as for cleavage variants of peptides activating BB_3_, GPR55, and GPR1 are shown in Table S7 and Figures S4 and S5.

**Figure S5:**
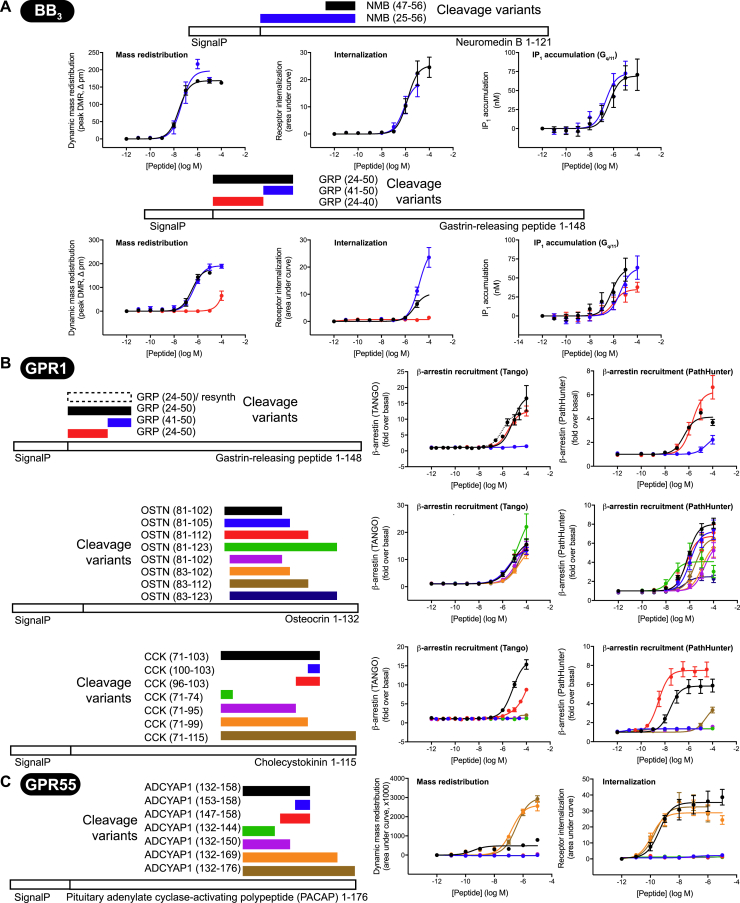
Peptide Ligand Variants Elicit Differential oGPCR Signaling Responses, Related to Figure 5 (A) BB_3_ responses for neuromedin B (NMB) and gastrin-releasing peptide (GRP) cleavage variants. (B) GPR1 responses for gastrin-releasing peptide, osteocrin (OSTN) and cholecystokinin (CCK-33) cleavage variants. (C) GPR55 responses for PACAP cleavage variants. Mass redistribution data show an apparently biphasic response for PACAP peptides, which could indicate an additional intracellular signaling pathway mediated via GPR55. Numbers in parenthesis indicate positions in the full-length protein.All data represent mean ± SEM for n = 3-4 independent experiments performed in triplicate.

We extensively characterized our peptide-GPCR receptor interactions using additional orthogonal G protein and/or β-arrestin assays (Figure 4B; Figure S4; Table S7). Our peptide-receptor pairing criterion was activity in at least two assays. Furthermore, we characterized additional cleavage variants of these peptides to improve their potency (Figure 5; Figure S5). These experiments also addressed the possibility that multiple peptide variants can be endogenous agonists (Tatemoto et al., 1998) and provided insights into determinants of activity for the discovered peptide ligands.

BB_3_ is an orphan receptor, although it responds weakly to physiologically relevant levels of the bombesin peptides neuromedin B and gastrin-releasing peptide, the endogenous agonists of BB_1_ and BB_2_, respectively (Alexander et al., 2017). We observed considerably more potent neuromedin B responses in mass redistribution (pEC_50_, 7.43 ± 0.08) and G_q/11_ signaling assays (IP_1_ generation; pEC_50_, 6.39 ± 0.42) (Figure 4B; Table S7) than reported previously (Jensen et al., 2008). We observed no differences in BB_3_ responses between the 10-amino acid neuromedin B peptide (designated NMB(47–56) based on its precursor residue number) and a longer, 32-residue cleavage variant, NMB(25–56), in any assay (Figure S5A; Table S7). We also found that the C terminus of gastrin-releasing peptide (GRP) activated BB_3_ with a potency comparable with the full-length peptide tested in the screen, whereas a truncated N-terminal variant, GRP(24–40), was less potent/efficacious or inactive. In addition to these ligands, we identified less potent BB_3_-mediated signaling for new cleavage variants derived from neuromedin-U and proenkephalin-A precursors (Figure 4B; Table S7). Taken together, these findings present multiple new peptide pairings for BB_3_, of which the neuromedin B peptides represent the most likely endogenous ligands for BB_3_, albeit at lower potency than at the BB_1_ receptor.

GPR1 (recently renamed chemerin receptor 2) has been reported as a chemerin receptor, although its primary biological function is currently unknown (Kennedy and Davenport, 2018). No G protein has been unequivocally linked with GPR1 (Figure S3). Accordingly, we observed robust and selective GPR1-dependent responses for four different peptides in two different β-arrestin recruitment assays but not in other assays (Figure S3, Figure S4A and S4A). Peptide 141 (Osteocrin_-2-19_) is the most potent (pEC_50_, 5.60 ± 0.15 in Tango and 6.18 ± 0.09 in PathHunter β-arrestin recruitment assays, respectively) (Table S7). Alternative osteocrin cleavage variants lacking two N-terminal amino acids had reduced potency (Figure S5B; Table S7). For another GPR1 hit, cholecystokinin (CCK-33), peptide cleavage variants were less active than the full-length peptide, except for a C-terminal 8-amino acid peptide, which was more potent in PathHunter β-arrestin recruitment assays than CCK-33 (Figure S5B; Table S7). We also found that gastrin-releasing peptide activated GPR1 in β-arrestin recruitment assays (Figure S5B; Table S7). Interestingly, we found that this activity was dependent on the peptide N terminus, whereas the C-terminal region GRP(41–50) was critical for BB_3_ signaling.

GPR15 was robustly activated by an 11-amino acid peptide derived from the C terminus of “*Uniprot:C10orf99”* (Figure 4B; Table S7). We demonstrated that GPR15 is G_i/o_-coupled because this peptide reduced cyclic AMP (cAMP) production (pEC_50_, 6.72 ± 0.13). Notably, two cleavage variants of this peptide (45 and 57 amino acids in length) had 10- and 100-fold improved potency, respectively (Figure 5; Table S7). In the course of our study, two other groups reported activation of GPR15 by the longest 57-residue cleavage variant, renamed GPR15L (Ocón et al., 2017, Suply et al., 2017). GPR15L contains two intramolecular disulfide bridges characteristic of CC family chemokines; however, it differs because peptide activity is not dependent on the N terminus (Ocón et al., 2017; Figure 5). Furthermore, we showed that the shortest, 11-residue C-terminal peptide, peptide 64 (GPR15L_47-57_) (lacking disulfide bridges) was sufficient to activate GPR15, although GPR15L represents the most potent and likely principal ligand.

We identified six peptides that promoted GPR55 internalization and mass redistribution, including five previously undescribed peptides and PACAP27, which exhibited a similar potency (pEC_50_, 9.51 ± 0.07) as for its cognate receptor, PAC_1_ (Alexander et al., 2017; Figure 4B; Figure S5C; Table S7). Progressive truncations of the PACAP-27 peptide completely abrogated the GPR55 response (Figure S5C; Table S7). Conversely, we found that longer peptide cleavage variants (PACAP-38 and a 45-amino acid variant) elicited similar picomolar-potency internalization responses to PACAP-27. One of the six GPR55 ligands, peptide 143 originates from rat hypothalamus (Secher et al., 2016), and testing of the human 143 confirmed activity, albeit with 10-fold lower potency (Table S7)

GPR68 is a proton-sensing receptor abundantly expressed in the hippocampus that is involved in learning and memory (Huang et al., 2015). We identified three peptides: 128 (Osteocrin_33-55_), 139 (CART(42-89)_9-28_), and rat 148 (Corticotropin_17-40_) that led to GPR68-dependent mass redistribution responses with sub- or low-micromolar potencies (Figure 4B; Table S7). These responses were confirmed in the real-time internalization assay, although with lower potency compared with mass redistribution or G protein signaling assays (Roed et al., 2014). We further demonstrated that these peptides activated G_q/11_ (calcium mobilization) and G_s_ (cAMP signaling) (Figure 4B; Figure S4A; Table S7), consistent with previously reported GPR68 signaling (Huang et al., 2015). We precluded a non-specific effect or direct proton-sensing mechanism of activity because none of these peptides induced responses in mock cells or elicited changes in extracellular pH (data not shown). Given the known allosteric modulation of proton-dependent GPR68 signaling by surrogate ligands, we performed further analyses of G_s_ signaling. These studies revealed that the three peptides are positive allosteric modulators of the proton responses, with up to ∼2-fold improved allosteric activity (log(αβ/K_B_)) over the small-molecule GPR68 ligand ogerin (Huang et al., 2015; Figure 4B; Figure S4A; Table S7). Taken together, our pairings represent the first peptides and the very intriguing examples of putative endogenous allosteric modulators of proton-dependent agonism at GPR68.

In addition, we identified new peptide pairings for five other orphan and understudied receptors in the β-arrestin recruitment assays: GPR17, GPR161, GPR176, GPR183, and MAS1 (Figure S4B; Table S7). Because these receptors were not among the 21 oGPCRs selected for the mass redistribution and receptor internalization assays, we did not pursue these findings here. We also identified nine “repeat hitters” (in more than 5 receptor-expressing or untransfected cells) that represent “orphan peptide ligands” for receptors not assayed here (Table S6). Furthermore, our complete library of 1,227 “cleavage variants” (Table S4) comprises a resource of putative ligands for future deorphanization. Finally, all of our “lead peptides” may be alternatively cleaved by carboxypeptidases or post-translationally modified into more potent biologically active receptor ligands, as for GPR15L (Figure 5; Table S7).

## Discussion

The discovery of ligand-GPCR signaling systems often translates into clinical opportunities but first requires independent validation and characterization by the wider research community. Hence, we sought to explore the disease associations of our peptide-receptor pairs and to independently validate previously proposed pairings.

### Therapeutic Potential of the Discovered Peptidergic Receptor Systems

**Figure S6:**
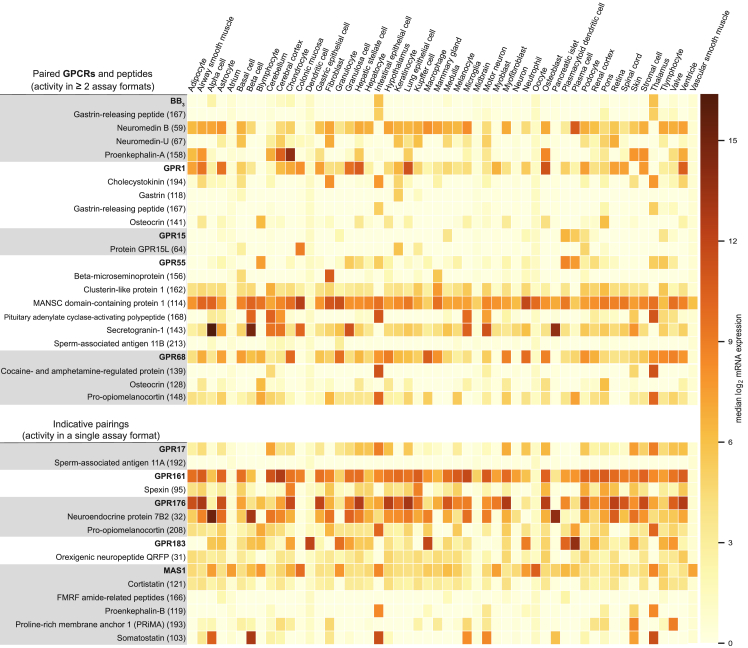
mRNA Expression Profiles of Paired Peptide Ligand Precursors and Receptors, Related to Figure 4 Human tissue expression profile of peptide-receptor pairs. Some receptors or precursors have low abundance (e.g., FMRF amide-related peptide) or restricted expression patterns (e.g., BB_3_, gastrin-releasing peptide), whereas the majority are ubiquitously expressed (source data from massive mining of publicly available RNA-seq data for 52 tissues provided by ARCHS4). Peptides are cleaved and secreted from their tissue of origin and may act at distant tissues.

**Figure 6:**
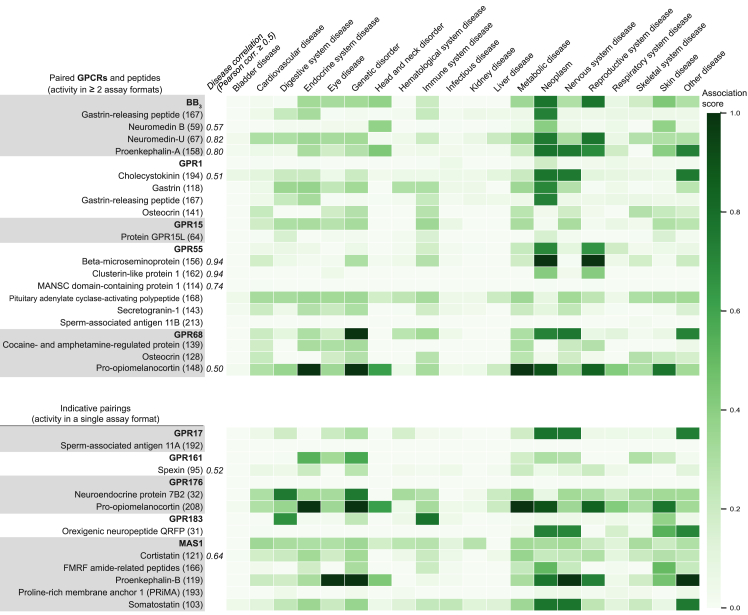
Disease Associations for Novel Peptide-Receptor Pairs Diseases associated with paired receptors and peptide precursors from https://www.opentargets.org. Open Targets presents associations with therapeutic areas by agglomerating data; e.g., genome-wide associations, genetic variants, expression and animal models. Disease association scores between 0 and 1 (color intensity) summarize the strength of evidence. For precursors with disease correlation similar to the associated receptor target, the Pearson correlation value is indicated. UniProt names for precursors are shown, with peptide library designation in parentheses (Table S4).

We combined literature reports with mRNA expression data from ARCHS4 (Lachmann et al., 2018; Figure S6) and disease associations from https://www.opentargets.org (Figure 6; Table S4; Koscielny et al., 2017). BB_3_ mouse knockout studies have demonstrated an important role in energy homeostasis, making this receptor a target in obesity and metabolic disorders (Alexander et al., 2017). Interestingly, the Open Target data present several additional disease areas (the strongest being nervous system disease) that strongly correlate with those of neuromedin-U and proenkephalin-A and moderately with neuromedin B (disease profile Pearson correlations of 0.82, 0.80, and 0.57, respectively; Figure 6).

GPR1 has been linked to cancer and cardiovascular and neurodegenerative disease (Kennedy and Davenport, 2018), which is reflected in a broad tissue expression profile. Osteocrin is a natriuretic peptide clearance (NPR3) receptor ligand implicated in bone and muscle function (Nishizawa et al., 2004, Thomas et al., 2003) and human brain development (Ataman et al., 2016). It has also been described as an endocrine hormone with potential therapeutic application to myocardial infarction (Chiba et al., 2017, Miyazaki et al., 2018).

GPR15 is expressed in immune cells, whereas GPR15L is expressed in epithelial cells. GPR15L is secreted during inflammation responses and therefore represents a promising target for inflammatory diseases, such as psoriasis and colitis (Suply et al., 2017). The Open Target database presents additional associations of GPR15 with diseases of the endocrine, genetic, metabolic, and nervous systems (Figure 6).

GPR55 responds to lipids, but the direct receptor dependence of this signaling is somewhat controversial (Alhouayek et al., 2018). The agonist-dependent GPR55 trafficking shown here for PACAP27 and five new peptides open further avenues to investigate its function. GPR55 is widely expressed and has been proposed as a potential therapeutic target for a range of diseases, including cancer, metabolic disorders, pain, and inflammation (Alhouayek et al., 2018). In Open Target, GPR55 and the precursors beta-microseminoprotein and clusterin-like protein 1 all have a strong link to neoplasm, supporting a potential link to cancer (Figure 6).

GPR68 acts as a proton sensor in bone, lung, and other tissue to regulate inflammatory responses, cell proliferation, and migration (Huang et al., 2015). Accordingly, it is a potential target in inflammation and cancer (and a secondary target in anxiety; Weiß et al., 2017). Recently, GPR68 has been described as a flow sensor in arteriolar endothelium involved in cardiovascular pathophysiology (Xu et al., 2018). Our GPR68 peptides span multiple therapeutic areas (Figure 6). Most notably, peptide 139 (CART(42-89)_9-28_) is a shorter variant of cocaine- and amphetamine-regulated portein (CART), which has been implicated in addiction (Kuhar, 2016). CART is an orphan peptide ligand of a GPCR; it has been shown to signal via protein kinase A, protein kinase C, and cAMP response element-binding protein (Chiu et al., 2009), as well as G_i/o_. Furthermore, the osteocrin-derived peptide 128 (Osteocrin_33-50_) shares disease associations with GPR68, spanning cardiovascular, eye, genetic, immune system, metabolic, and nervous system diseases. Finally, the pro-opiomelanocortin-derived peptide 148 (Corticotropin_17-40_) and GPR68 both have strong associations with genetic and neoplastic disease. These analyses suggest that GPR68 may hold (patho)physiological roles beyond proton and flow sensing. Moreover, the three peptides act as positive allosteric modulators of the proton responses (Figure 4B; Figure S4A; Table S7), suggesting that the two types of ligands may act in concert to regulate GPR68 activity.

### Confirmation of Proposed Pairings and Identification of Secondary Ligands Expand the Peptide GPCR Network

**Figure S7:**
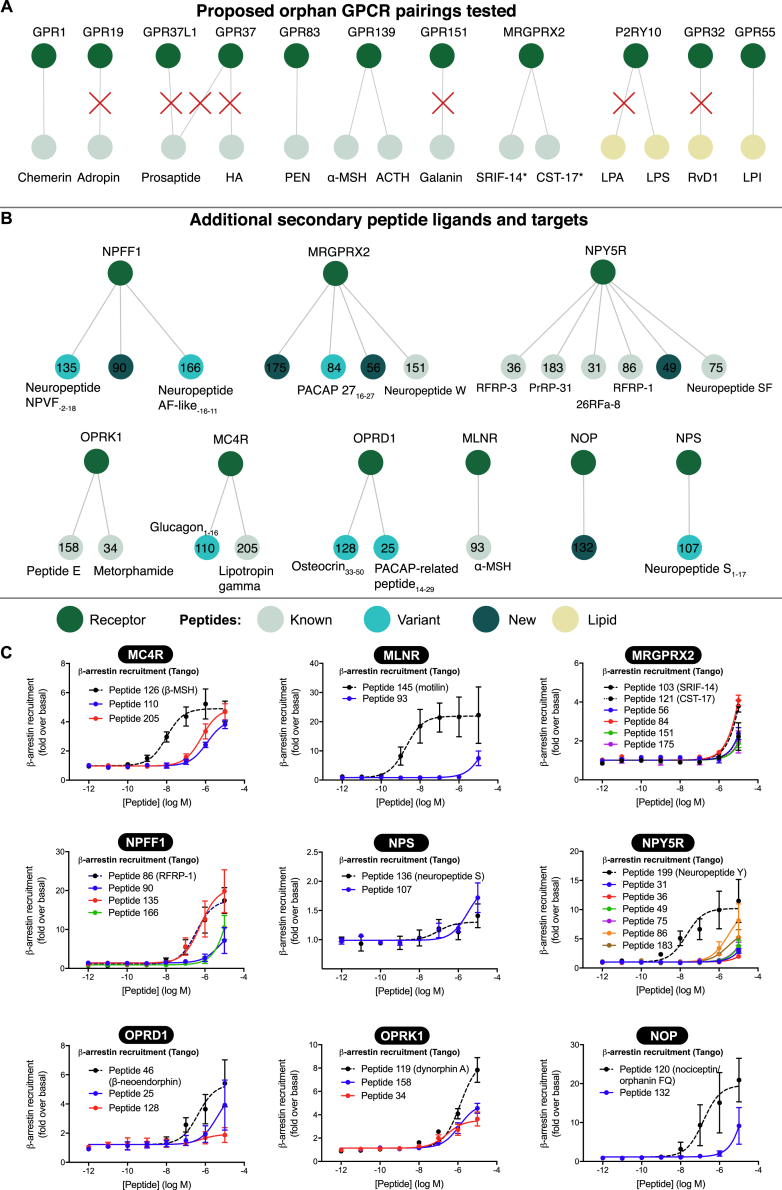
Confirmation of Proposed Pairings and Secondary Ligands for Known Peptide Receptors, Related to Figure 7 (A) Of the 14 pairings proposed in literature, half were reproduced in mass redistribution and/or internalization assays (Table S5). Note: P2RY10–lysophosphatidylserine (LPS) activity was marginal. ^∗^We tested cleavage variants of cortistatin (CST-14) and somatostatin (SRIF-28). (B-C), Nine known peptide receptors were activated by their cognate agonists (internal controls) and, unexpectedly, 22 additional peptides. These indicate as yet unappreciated cross-pharmacology (Table S7). All data represent mean ± SEM for n = 3 independent experiments performed in triplicate.

Independent confirmation of proposed ligand-receptor pairings promotes consensus in the field and is essential to choose and design the optimal follow-up studies (Davenport et al., 2013). Consensus regarding pairings and their physiological relevance is collated in the Guide to Pharmacology database (Harding et al., 2018). We sought to repeat literature pairings for our 21 predicted peptide receptors (Figure S7A). We confirmed previously proposed pairings of chemerin/GPR1, the neuroendocrine peptide PEN/GPR83 (Gomes et al., 2016), the melanocortin receptor ligands α-MSH and ACTH/GPR139 (Nøhr et al., 2017), cortistatin and somatostatin/MRGPRX2, LPI/GPR55 (Henstridge et al., 2010), and LPS/P2RY10 (Inoue et al., 2012) by selective receptor-dependent activation across multiple assays (Tables S5 and S7). In contrast, we found no activity for other proposed peptide/GPCR pairings such as adropin/GPR19 (Stein et al., 2016), head activator/GPR37 (Rezgaoui et al., 2006), prosaptide/GPR37/GPR37L1 (Meyer et al., 2013, Rezgaoui et al., 2006), and galanin/GPR151 (Ignatov et al., 2004) or for the lipid/GPCR pairings resolvin D1/GPR32 (Krishnamoorthy et al., 2010) and lysophosphatidic acid/P2RY10 (Murakami et al., 2008) (Tables S5 and S7). The majority of proposed pairings evaluated here were not assessed in previously published large orphan receptor screening studies, which measured β-arrestin recruitment in PathHunter (Southern et al., 2013) or Tango assays (Kroeze et al., 2015).

Intriguingly, we also found that nine class A peptide receptors are activated by six previously published and 16 potential new peptide ligands (Figures S7B and S7C; Table S7). This included a truncated glucagon variant that stimulated the melanocortin MC_4_ receptor and a prolactin-releasing peptide variant that activated NPY_5_. Although the potency of these peptides is lower than for their principal agonists, the potential secondary/cross-pharmacology warrants further investigation.

**Figure 7:**
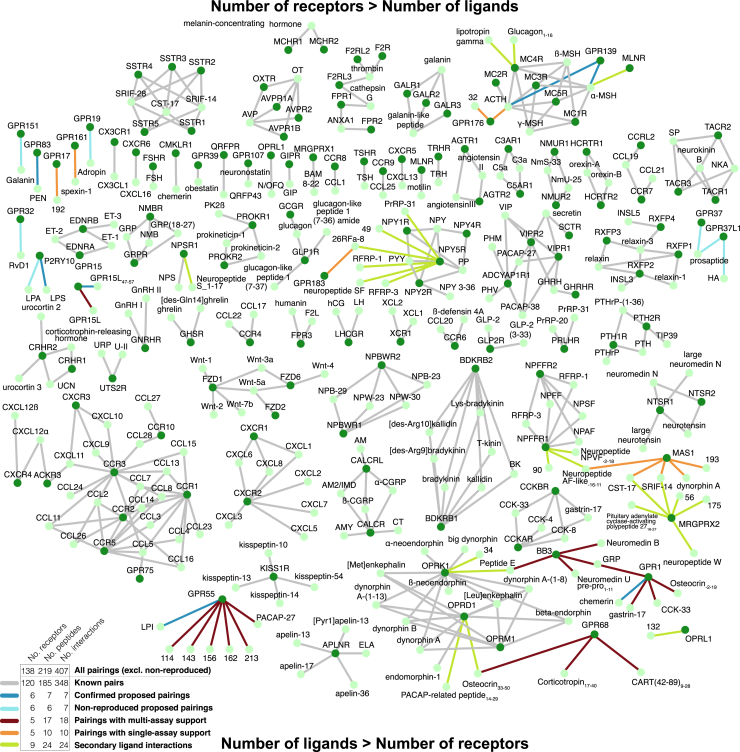
Expansion of the Human Peptidergic Receptor Signaling System The new pairings (colored lines) increase the number of known ligand-receptor connections (edges) from 348 to 407 (putative peptide ligands from 185 to 214 and putative peptide receptors from 120 to 130). Ligand-receptor systems are shown with increasing ligand-to-receptor ratios (top to bottom). There are more ligands per receptor in both the established and novel peptidergic receptor systems.

In conclusion, our combined computational and pharmacological approach has expanded the known human peptidergic signaling network from 348 to 407 interactions (an increase of 17%; Figure 7). 39 (74%) of the 53 peptides with validated receptor-dependent responses were first discovered here, demonstrating the predictive power of our approach, which could be transferred to many other peptide/protein systems. The discovery of peptide ligands for GPCRs has previously opened fields of research and is often closely followed by rapid translation into the clinic. Therefore, our findings are expected to fuel many future studies to establish their physiological roles and therapeutic potential.

## STAR★Methods

### Key Resources Table

**Table:** 

REAGENT or RESOURCE	SOURCE	IDENTIFIER
**Antibodies**		

Monoclonal anti-FLAG M2	Sigma Aldrich	Cat # F1804; RRID: AB_262044
HRP labeled anti-Mouse IgG	Vector Laboratories	Cat # PI-2000; RRID: AB_2336177

**Chemicals, Peptides, and Recombinant Proteins**		

Dulbecco’s modified Eagle’s medium (DMEM) without pyruvate	Thermo Fisher Scientific	Cat # 61965026
Dialyzed FBS	Thermo Fisher Scientific	Cat # 26400036
Doxycycline hyclate	Sigma Aldrich	Cat # D9891
Zeocin	Thermo Fisher Scientific	Cat # R25001
Blasticidin	Thermo Fisher Scientific	Cat # A11139-03
Hygromycin B	Thermo Fisher Scientific	Cat # 10687010
Lipofectamine 2000 Transfection Reagent	Thermo Fisher Scientific	Cat # 11668027
Polyethylenimine (PEI), Linear, MW 25000, Transfection Grade	Polysciences	Cat # 23966
Poly-L-lysine	Sigma Aldrich	Cat # P2636
SNAP-Lumi4-Tb (terbium)	Cisbio	Cat # SSNPTBD
Fluorescein-O′-acetic acid	Sigma Aldrich	Cat # 88596
Fluo-4 AM	Invitrogen	Cat # F14201
Probenecid	Invitrogen	Cat # P36400
MES, 2-(N-morpholino)ethanesulfonic acid	Alfa Aesar	Cat # H56472
HEPES, 4-(2-hydroxyethyl)-1-piperazineethanesulfonic acid	Fisher Scientific	Cat # BP310
TAPS, (tris(hydroxymethyl)methylamino)propanesulfonic acid	VWR	Cat # VWR J562
Ro 20-1724	Cayman Chemical	Cat #18272
Luciferin	Goldbio	Cat # LUNCA
DMEM	Corning	Cat #10-013-CM
Dialyzed FBS	Omega Scientific	Cat # FB-03

**Critical Commercial Assays**		

SuperSignal ELISA Femto Substrate	Thermo Fisher Scientific	Cat # 37075
Bright-Glo Luciferase assay system	Promega	Cat # E2620
IP-One - Gq kit	Cisbio	Cat # 62IPAPEC
cAMP - Gs Dynamic kit	Cisbio	Cat # 62AM4PEJ

**Deposited Data**		

Crystal Structure of Bovine Rhodopsin at 2.2 Angstroms Resolution	Protein Data Bank	PDB: 1U19
High resolution crystal structure of human B2-adrenergic G protein-coupled receptor.	Protein Data Bank	PDB: 2RH1
The 2.5 A structure of the CXCR4 chemokine receptor in complex with small molecule antagonist IT1t	Protein Data Bank	PDB: 3ODU
Structure of the human dopamine D3 receptor in complex with eticlopride	Protein Data Bank	PDB: 3PBL
Structure of the human histamine H1 receptor in complex with doxepin	Protein Data Bank	PDB: 3RZE
Structure of the human M2 muscarinic acetylcholine receptor bound to an antagonist	Protein Data Bank	PDB: 3UON
Crystal Structure of a Lipid G protein-Coupled Receptor at 2.80A	Protein Data Bank	PDB: 3V2Y
High Resolution Structure of Thermostable Agonist-bound Neurotensin Receptor 1 Mutant without Lysozyme Fusion	Protein Data Bank	PDB: 4BUO
Ultra-thermostable beta1-adrenoceptor with cyanopindolol bound	Protein Data Bank	PDB: 4BVN
Structure of the human kappa opioid receptor in complex with JDTic	Protein Data Bank	PDB: 4DJH
Crystal structure of the mu-opioid receptor bound to a morphinan antagonist	Protein Data Bank	PDB: 4DKL
1.8 A Structure of the human delta opioid 7TM receptor	Protein Data Bank	PDB: 4N6H
M3-mT4L receptor bound to tiotropium	Protein Data Bank	PDB: 4U15
Crystal Structure of Human Lysophosphatidic Acid Receptor 1 in complex with ONO-3080573	Protein Data Bank	PDB: 4Z36
Structures of the human OX1 orexin receptor bound to selective and dual antagonists	Protein Data Bank	PDB: 4ZJ8
Crystal Structure of Human Angiotensin Receptor in Complex with Inverse Agonist Olmesartan at 2.8A resolution	Protein Data Bank	PDB: 4ZUD
Structure of the human M1 muscarinic acetylcholine receptor bound to antagonist Tiotropium	Protein Data Bank	PDB: 5CXV
The crystal structure of nociceptin/orphanin FQ peptide receptor (NOP) in complex with SB-612111	Protein Data Bank	PDB: 5DHH
Structure of the M4 muscarinic acetylcholine receptor (M4-mT4L) bound to tiotropium	Protein Data Bank	PDB: 5DSG
Crystal structure of the human CC chemokine receptor type 9 (CCR9) in complex with vercirnon	Protein Data Bank	PDB: 5LWE
A2A Adenosine receptor room-temperature structure determined by serial femtosecond crystallography	Protein Data Bank	PDB: 5NM4
High-resolution crystal structure of the human CB1 cannabinoid receptor	Protein Data Bank	PDB: 5U09
Crystal structure of the human adenosine A1 receptor A1AR-bRIL in complex with the covalent antagonist DU172 at 3.2A resolution	Protein Data Bank	PDB: 5UEN
Crystal Structure of CC Chemokine Receptor 5 (CCR5) in complex with high potency HIV entry inhibitor 5P7-CCL5	Protein Data Bank	PDB: 5UIW
Structure of apelin receptor in complex with agonist peptide	Protein Data Bank	PDB: 5VBL
Structure of the human D4 Dopamine receptor in complex with Nemonapride	Protein Data Bank	PDB: 5WIU
Crystal structure of human orexin 2 receptor bound to the selective antagonist EMPA determined by the synchrotron light source at SPring-8.	Protein Data Bank	PDB: 5WQC
Human endothelin receptor type-B in complex with antagonist K-8794	Protein Data Bank	PDB: 5X93

**Experimental Models: Cell Lines**		

Flp-In T-REx 293 cells	Thermo Fisher Scientific	Cat # R78007; RRID: CVCL_D585
Orphan GPCR T-REx 293 cell lines	This study	N/A
HTLA cells	Gift from G. Barnea and R. Axel (Brown University and Columbia University)	N/A
HA-OGR1 (GPR68) HEK293 cells	Saxena et al., 2012	N/A
pcDNA3 (mock) HEK293 cells	Saxena et al., 2012	N/A
293T cells	ATCC	Cat # CRL-3216; RRID: CVCL_0063

**Recombinant DNA**		

pcDNA5/FRT/TO Vector Kit	Thermo Fisher Scientific	Cat # V652020
pOG44 Flp-Recombinase Expression Vector	Thermo Fisher Scientific	Cat # V600520
pcDNA6/TR	Thermo Fisher Scientific	Cat # V102520
pcDNA5/FRT/TO FLAG SNAP	Pedersen et al., 2019	N/A
Roth Lab PRESTO-Tango GPCR Kit	Kroeze et al., 2015	Addgene Kit # 1000000068
GloSensor cAMP plasmid	Promega	Cat # E2301

**Software and Algorithms**		

Graphpad Prism 7	Graphpad Software	https://www.graphpad.com/
Masshunter	Agilent	
PyMOL Molecular Graphics System, Version 2.0	Schrödinger	https://pymol.org/2/; RRID:SCR_000305
Bio3D v2.3	Skjærven et al., 2016	http://thegrantlab.org/bio3d/index.php
Arpeggio		http://biosig.unimelb.edu.au/arpeggioweb/
Pandas v0.20.3	Wes McKinney	https://pandas.pydata.org/
Scikit-learn v0.19.2	scikit-learn community	https://scikit-learn.org/
CD-HIT	Huang et al., 2010	http://weizhongli-lab.org/cd-hit/
GPCRdb	Pándy-Szekeres et al., 2018	https://github.com/protwis/protwis
Python v2.7.13 and v3.6.5	Python Software Foundation	https://www.python.org/; RRID:SCR_008394
GetContacts	Rasmus Fonseca and Anthony Ma	https://getcontacts.github.io/
Flareplots	Rasmus Fonseca	https://gpcrviz.github.io/flareplot/
bio2mds	Pelé et al., 2012	https://cran.r-project.org/web/packages/bios2mds/index.html
Rate4Site	Pupko et al., 2002	https://www.tau.ac.il/∼itaymay/cp/rate4site.html
Computed Atlas of Surface Topography of proteins (CASTp) 3.0	Tian et al., 2018	http://sts.bioe.uic.edu/castp/index.html?2r7g
Theseus	Theobald and Steindel, 2012	https://theobald.brandeis.edu/theseus/
Maestro Schrödinger Release 2017-4	Schrödinger	https://www.schrodinger.com/maestro
Custom scripts for analysis	This study	Available upon request

**Other**		

Epic Benchtop (BT) System	Corning	Cat # 5053
Epic 384-well cell assay microplate, fibronectin-coated	Corning	Cat # 5042
EnVision multimode plate reader	PerkinElmer	Cat # 2104
EnSpire multimode plate reader	PerkinElmer	Cat # 2300
Flex Station III	Molecular Devices	Cat # FLEX3

### Lead Contact and Materials Availability

Further information and requests for resources and reagents should be directed to and will be fulfilled by the Lead Contact, David E. Gloriam (david.gloriam@sund.ku.dk). The 21 orphan receptor cell lines generated in this study are available from the Lead Contact with a completed Materials Transfer Agreement.

### Experimental Model and Subject Details

#### Mammalian cell culture conditions

Flp-In T-REx 293 Cells (Thermo Fisher Scientific) were used for the generation of all oGPCR stable cell lines that were used in dynamic mass redistribution (DMR) and receptor internalization screening, as well as subsequent cell signaling assays. This system is of particular utility for studies on orphan receptors, where heterologous overexpression may be toxic to the cells over prolonged periods (Bercher et al., 2009), or where the potential endogenous ligand may be present in the culture media. Cells were grown at 37°C and 5% CO_2_ in Dulbecco’s modified Eagle’s medium (DMEM) with 4.5 g/L glucose and GlutaMAX Supplement, without pyruvate (GIBCO), supplemented with 10% dialyzed FBS (Thermo Fisher Scientific), 100 U/mL penicillin-streptomycin and 15 μg/mL blasticidin (complete medium; Thermo Fisher Scientific). Prior to transfection with SNAP-tagged receptor constructs, parental Flp-In T-REx 293 cells were cultured in complete medium supplemented with 100 μg/mL zeocin (Thermo Fisher Scientific). oGPCR transfections were performed using Lipofectamine 2000 (3 μL of Lipofectamine per 1 mg of DNA). All stable cell lines were selected and maintained in complete medium supplemented with 200 μg/mL hygromycin B (Thermo Fisher Scientific). HTLA cells were a gift from the laboratory of R. Axel and were maintained in DMEM (Corning) supplemented with 10% FBS, 100 U/mL penicillin and 100 μg/ml streptomycin, 2 μg/mL puromycin and 100 μg/mL hygromycin B in a humidified atmosphere at 37°C in 5% CO_2_. HEK293T and HEK293 stable cell lines (HA-OGR1 (GPR68) and vector pcDNA) (Saxena et al., 2012) were cultured at 37°C with 5% CO_2_ to near confluence in DMEM with 10% FBS (Corning).

### Method Details

#### Identification of human endogenous peptide and small molecule sets

To identify all human genome encoded peptides, we extracted the human proteome annotation from UniProtKB/Swiss-Prot (version 2018.3; released 28/03/2018) and searched for all *PEPTIDE* annotations. This led to a unique set of 163 peptide precursor proteins and a total annotated set of 378 peptides, in many instances including multiple variants of similar peptides. For instance, apelin is represented in four versions including apelin-13, apelin-28, apelin-31 and apelin-36. In an orthogonal approach, we extracted all endogenous ligand entries including peptides and metabolites from the International Union of Basic and Clinical Pharmacology/British Pharmacological Society (IUPHAR/BPS) Guide to Pharmacology database (version 2018.1; released 05/03/2018) (Harding et al., 2018) using custom Python scripts. Endogenous peptides were defined as ligands originating from within the studied organism shown to have activity at the receptor. Small molecules were defined by IUPHAR (“Metabolites”) as low molecular weight, non-peptide, biogenic compounds produced by life processes and their close analogs. Of the 341 endogenous human peptides, 230 target GPCRs (67.4%) and 185 are primary peptides annotated as “principal” endogenous ligands for the target (e.g., including angiotensin II and III for the angiotensin II type-1 receptor (AT_1_), but not angiotensin A and IV). Of the 174 endogenous human metabolites, 112 are known to target GPCRs (64.4%) and 76 are primary metabolites that are annotated as main endogenous activators. For each endogenous ligand, molecular weight was calculated using *Molmass*. Affinity and potency (pKi, pKd, pEC_50_ and pIC_50_) values were obtained from the Guide To Pharmacology database. When multiple potency/affinity values were provided, we used the maximum reported.

#### Evolutionary analysis of peptide receptor and peptide ligand repertoires

##### Determination of receptor and ligand repertoires

The set of 42 known human annotated peptide GPCR/ligand-precursor families (across classes) consisting of 120 receptors and 130 ligand precursors, was obtained from the IUPHAR/BPS Guide to Pharmacology database (version 2018.1; released 05/03/2018) (Harding et al., 2018). To determinate sequence relationships of receptor-ligand families across different organisms, we collected phylogenetic relationships and orthologous sequences through the REST API from the Orthologous MAtrix (OMA) database (version December 2017) (Altenhoff et al., 2015) using R and Python scripts written in-house. UniProtKB/Swiss-Prot identifiers were used for cross-platform ID mapping (UniProt Consortium, 2018). Given these identifiers, OMA, which uses the human genome from Ensembl, had ortholog data for 114/115 human peptide GPCRs and for 118/120 peptide precursors. For missing proteins, the closest homologs of identical gene names were used (a list of missing receptors and alternative IDs are given as Table S2). Ortholog data was retrieved from all 313 available eukaryotic organisms (Taxon ID: 2759). To determine the true size of each receptor-ligand family system (and to cover possible gene duplication events) in a given species, we investigated all in-paralogs for every ortholog in a given species. For this analysis, all pairwise protein relationships were extracted for proteins within a species and to the human reference. This led to a total 23,606,407 pairwise protein relationships across 313 eukaryotic organisms. We calculated the Jaccard similarity index to determine the similarity of a given receptor-ligand family repertoire between a species and human. The Jaccard similarity index (range 0–1), was defined as the number of conserved genes (overlapping) divided by the total number of genes that code for receptors or ligands in a specific family, respectively. To identify the overlap of the receptor and ligand repertoires in different organisms, genes in different organisms were annotated as having an overlapping phylogenetic relationship if they had a OMA reported 1:1 or 1:*n* relationship (OMA definition: “*The entry has more than one ortholog in the other species but all orthologous entries have only one ortholog in this species. This implies that the gene was duplicated in an ancestor of the other species, but after the speciation event*”). Hence, species unique proteins were identified by all in-paralogs without any 1:1 or 1:*n* relationship to human. A high Jaccard index (closer to 1) means that the two organisms largely share the receptor-ligand repertoire for this family. A lower value (closer to 0) means that the repertoires are more distinct. A large number of distinct genes in the organism for which orthologs do not exist in humans suggests that this lineage has undergone independent expansion of the receptor or ligand repertoire through gene duplication events. To investigate receptor ligand repertoire similarity coevolution, we calculated the Pearson product-moment correlation coefficient as a statistical measure of the correlation (linear association) between the ligand and receptor Jaccard similarity indices. The gene numbers provided here offer an update to previous estimates of the receptor-ligand repertoire in some of these organisms.

##### Calculation of receptor-ligand fingerprints

We made use of the large number of available complete DNA genome sequences and employed phylogenetic profiling for all receptor/ligand families by exploring the joint presence or joint absence of two genes (Pellegrini et al., 1999). An evolutionary fingerprint was generated for each protein by searching for its 1:1 ortholog among all OMA available eukaryotic organisms (n = 313). The fingerprint was recorded as a binary code in which the presence or absence of an ortholog is registered as “1” (positive bit) or “0” (negative bit), respectively. Based on the assumption that receptor and ligands co-evolve and therefore should have orthologs in closely related species, we expected more similar fingerprints for receptor-ligand pairs than for random protein-protein pairs. In order to probe this assumption, we calculated fingerprint similarities for known receptor-ligand pairs. The similarity was calculated as the ratio of fingerprint bit mismatches to the length of the total fingerprint:

$$\mathrm{similarity}\left(\%\right)=\frac{\mathrm{identical fingerprint bits}}{\mathrm{total fingerprint bits}}\times 100$$

Enrichment of all 238 known unique receptor/ligand-precursor pairs was assessed with permutation tests by performing 10,000 randomization trials. In each randomization, each ligand was replaced with a random gene of several categories and the fingerprint similarity was noted. From the random distribution, we computed the Z-score, which captures the distance of the actual fingerprint similarity (e.g., the known receptor-ligand pair) to the mean of random expectation in terms of the number of standard deviations. We estimated the *p* value as the ratio of the number of simulations where the random observations were greater than or equal to the number of observed values to the total number of randomizations (10,000). Random selection was performed for the full proteome (n = 20,234 proteins), secreted proteins as annotated in UniProt (n = 2,753), or essential cell genes (deemed essential in multiple cultured cell lines based on shRNA screen data) (Hart et al., 2014). A random selection among the 16 G proteins was performed as a positive control.

To deconvolute the strongest signal of fingerprint correlations, different merging strategies of receptor and/or ligand fingerprints were investigated. Briefly, we merged the fingerprints of different sets by summation of all positive bits (‘1 s’) in an aligned fingerprint. For instance, when merging receptor fingerprints, we measure the presence of an ortholog for a given species if an ortholog was reported in at least one of the to-be-merged receptors. Likewise, we merged fingerprints of ligands within the same receptor family (e.g., chemokine receptors; ligand family merge) and fingerprints of ligands binding to the same receptor (ligand binding merge).

#### Peptide receptor analysis

##### Analysis of loop lengths

All GPCR receptor sequences were obtained using the Web-services of GPCRdb (Pándy-Szekeres et al., 2018) including structure-based GPCRdb numbering as an adaption of the sequence-based Ballesteros-Weinstein scheme with corrections for helix bulges and constrictions (Isberg et al., 2015). Length comparisons were made between the most conserved transmembrane (TM) residues, as well as for the extracellular loop 2 (ECL2) between 4x50 and 5x50 and for ECL3 between 5x50 and 6x50 according to the GPCRdb numbering scheme. ECL2 was further separated into the region before and after the conserved cysteine (C45x50; conserved in 87% of all class A and 83% of all GPCRs). For each (class A) non-orphan receptor, the length of that region was calculated and normalized according to the different receptor families to avoid the over representation of large families (family sizes range from 1 – 23 members). For each receptor family, the median segment length was obtained. We estimated statistical significance of differences in the distribution of receptor loop lengths of GPCRs using the non-parametric Wilcoxon rank sum test.

##### Analysis of β sheets in crystal structures

All available peptide and protein receptor structures were retrieved from the PDB and visualized using PyMOL (The PyMOL Molecular Graphics System, version 2.0 Schrödinger, LLC.) comprising of 17 and 5 unique crystallized receptors for peptide and protein binding GPCRs, respectively. Structural superpositioning was performed with *Theseus* (Theobald and Steindel, 2012). All peptide and protein structures with resolved ECL2 contain a β sheet, whereas no β sheet was observed for other crystal structures. To further investigate potential characteristic structural features, we compared the sequence alignments of the ECL2 region comprising the β sheet. As no generic structure based-alignment positions exist for most of the ECL2, we constructed an alignment anchored on the conserved CYS^45x50^. The residue number for every GPCRs 45x50 position was recalled and all consecutive residues were aligned without gaps. A difference matrix of frequencies of each amino acid for these positions was created for the peptide/protein receptors and other class A receptor sets.

##### Principal component analysis of protein structures

Biostructural analysis of Class A GPCR structures was conducted using *Bio3D* (Skjærven et al., 2016). A representative set of 28 unique inactive receptor structures (13 peptide/protein; 15 aminergic, nucleotide, sensory and lipid receptors) was compiled, using the highest resolution structure for each available receptor structure. Peptide/protein structures and chains were:PDB: 4N6H_A, PDB: 5WQC_A, PDB: 5UIW_A, PDB: 5X93_A, PDB: 3ODU_A, PDB: 5VBL_B, PDB: 4ZJ8_A, PDB: 4BUO_A, PDB: 5LWE_B, PDB: 4ZUD_A, PDB: 4DKL_A, PDB: 4DJH_A, PDB: 5DHH_A. Non-peptide/protein structures in Class A and associated chains were: PDB: 5NM4_A, PDB: 5WIU_A, PDB: 4BVN_A, PDB: 1U19_A, PDB: 2RH1_A, PDB: 5DSG_B, PDB: 5U09_A, PDB: 5CXV_A, PDB: 3V2Y_A, PDB: 4U15_B, PDB: 3PBL_A, PDB: 4Z36_A, PDB: 3UON_A, PDB: 3RZE_A, PDB: 5UEN_B. Structures were superposed on 38 invariant core positions. Principal component analysis (PCA) was used to provide a two-dimensional representation of the superposed structure set to summarize inter-conformer relationships (Skjærven et al., 2014). PCA revealed that 45.5% of the total coordinate variance could be captured in the first three dimensions (PC1-3). The first two principal components have been projected onto the principal planes in a conformer plot. PC1 and PC2 have been clustered with the *ward.D2* method in a cluster dendrogram that can be partitioned into 3 cluster groups. Peptide/protein structures separate into distinct and prominent clusters that are distant from other structures. Displacement trajectories of PC1 and PC2 showed most residue displacement from the mean structure for the upper TM regions of TM1 to TM5 including ECL1 and ECL2, which accounts for the majority of the difference between the cluster groups.

##### Comparison of binding site cavities

The representative list of highest resolution structure for each available class A receptor (as described above - *Principal component analysis of protein structures*) was selected and prepared with the protein preparation wizard in Maestro (SiteMap, Schrödinger Release 2017-4) using default settings after removal of all ligands in the orthosteric binding site. Buried ligand-binding pocket areas and volumes have been calculated and inspected using the *Computed Atlas of Surface Topography of proteins* (CASTp) with a probe sphere of 1.4 Å (Tian et al., 2018).

##### Multi-dimensional scaling of sequence signatures

To investigate sequence signatures of class A receptors, we performed Multi-Dimensional Scaling (MDS) using the *bio2mds* package in R (Pelé et al., 2012). Metric MDS is a statistical analysis technique aimed at analyzing a matrix of distances between ‘active’ elements. First, a multiple sequence alignment (MSA) of all ligand-interacting class A generic residue positions (n = 68) was constructed. Given the MSA as an input, a distance matrix of pairwise distances between sequences based on sequence dissimilarity was computed. The amino acid substitution matrix was set to Jones, Taylor and Thornton mutation data matrix for transmembrane proteins (JTT_TM) – a transmembrane protein exchange matrix (Jones et al., 1994). The MDS coordinates were then mapped onto a low dimensional (2D) space. Most peptide/protein receptors cluster apart from other ligand type families. Aminergic receptors cluster most prominently from other clusters for the first two principal components (∼17% of the total coordinate variance).

##### Selection of putative peptide orphan receptors

The selection of orphan receptor targets in this study was focused on putative peptide receptors, as peptide ligands could be identified from evolutionary analysis using bioinformatics. All approaches were based on the assumption that peptide receptors have distinct structural features reflected in their protein sequences, which accommodate the binding of peptide ligands that are generally more complex than small molecules, due to greater size and conformational freedom.

Comprehensive analysis of known class A peptide receptors revealed that there are common sequence and structural characteristics, making distinct classifications from non-peptide GPCRs possible. These peptide receptor-specific features include a prolonged ECL2, a conserved β sheet structure in ECL2 with a β turn-inducing sequence motif and a distinct sequence signature, which presumably combine to enlarge the upper TM binding pocket and to promote peptide ligand recognition.

More specifically, consensus peptide receptor family sequences were compared to non-peptide receptor families for every generic position based on the GPCRdb numbering scheme. Comparisons of every single position revealed 17 generic residue positions with at least 40% difference in one of the physico-chemical properties between peptide and non-peptide GPCRs. These over-represented residue positions tended to be mostly in the upper half of the TM region that is involved in ligand binding. The 17 residue positions were used to build a logistic regression model, which was then utilized to predict the likelihood of a given orphan receptor amino acid sequence being closer to peptide or non-peptide receptors, respectively. A retrospective analysis using this model enabled highly significant differentiation between peptide and non-peptide receptor families (logistic regression; *p* value < 0.0001).

These data indicated that although peptide families lack a shared phylogenetic relationship, peptide binding receptors can be identified based on intrinsic structural and functional sequence characteristics. However, not all peptide receptors share all of the investigated features (see a full ranking of all class A oGPCRs provided in Table S4). Therefore, a combined prediction model and individual selection was used to classify orphan receptors as peptide binding of which 21 receptors have been selected to assay and screen for endogenous peptide ligands.

#### Peptide ligand analysis and library design

##### Peptide lengths, positions and cleavage sites

Peptide sequences were obtained from IUPHAR and queried within the precursor protein sequence obtained from UniProtKB to identify starting positions and flanking regions. Most peptides could be readily mapped, although there were several exceptional cases with additional, alternative or unknown maturation processes. For instance, prokineticin-2β is a variant peptide that is 21 amino acids shorter than the parent peptide that is believed to arise as the result of post-processing protein cleavage events. Some peptide ligands (n = 18; ∼5%) such as the glycoprotein hormones (consisting of two chains that assemble to the biologically active heterodimeric hormone), the relaxin ligands (heterodimers linked by two disulfide bonds) and proteinase-activated receptors require more complex maturation. Surprisingly, the gene that encodes the precursor of endomorphin-1 has still not been identified (Terskiy et al., 2007). These exceptions indicate that our library design approach cannot cover all potentially encoded peptides.

The flanking regions of mapped peptide sequence have been identified for 184 peptides. Of these, 92 out of the 124 “principal” mature peptides of single chain ligands start immediately following the signal peptide and end at the final residue of the preproprotein. Of the remaining peptides, approximately 80% are C-terminally flanked by a cleavage motif consisting of any combination of dibasic amino acids (KR, RR, RK, KK plus GR or GK) and 66% are N-terminally flanked by a dibasic amino acid cleavage motif. The majority of the cleaved peptides have larger “pre-” processing versions or known alternative cleavage mechanisms, such as kallidin cleavage by kallikrein or C3 by C3 convertase. Cleavage motifs were shown as % difference by IceLogo, which is the increased frequency of the amino acid at the indicated relative position compared to a background comparison. The average length of peptide ligand precursors was shown to be 179 amino acids compared to the median human protein length of 375 amino acids. The average/median length of peptide ligands was observed to be 56.9 and 32.0 amino acids, respectively.

##### Filtering the human proteome for candidate precursors

Potential peptide ligand precursors were identified from the Swiss-Prot reviewed human proteome as those that were annotated as secreted or possessed a signal peptide. We specifically excluded precursors based on Gene Ontology (GO) terms such as “antioxidant activity,” “catalytic activity” and others based on GO annotations observed from known peptide precursors to focus on proteins with unknown molecular function (see Table S4). Additionally, we filtered precursors of more than 750 amino acid length. This led to a total set of 1,422 potential precursors for which we performed an evolutionary trace analysis and cleavage site mapping.

##### Ortholog identification

A widely used method for the detection of putative orthologous genes is that of Reciprocal Best Hits (RBH), where two genes from two different genomes are considered orthologs if their protein products find each other as the best hit in the opposite genome (Rivera et al., 1998). Briefly, the input sequence is matched to the human Swiss-Prot proteome using blastp, which compares a protein sequence to a protein database. The best hit is regarded as the human protein used to query against the target organism’s proteome by blastp. The top hit protein sequence is then in return queried back to the human proteome using blastp. If the resulting top hit human protein is the same protein as the initially identified human protein, the genes are considered orthologs. RBH was implemented using custom Python scripts. For candidate 1,422 potential precursors were subjected to ortholog queries in 74 organisms.

##### Evolutionary trace analysis

Multiple sequence alignment (MSA) of the orthologs for each precursor was performed using MUSCLE with default settings (Edgar, 2004). The relative amino acid substitution evolutionary rate of each position was calculated using Rate4Site (R4S) version 3.0.0.2 (Pupko et al., 2002). Rate4Site assigns a per-site variability score that indicates how rapidly each residue evolves relative to the mean protein rate for each position in the MSA using an empirical Bayesian inference. Evolutionary trace profiles were generated for 1,392 precursor genes. Analysis and visualizations were performed using custom Python scripts using *pandas* and *matplotlib* packages.

##### Generation of peptide library

For each potential precursor gene with evolutionary trace information (n = 1,392) all potential peptides after the signal peptide of 3-45 amino acids length between conserved cleavage sites (see Peptide lengths, positions and cleavage sites) or the C terminus were retrieved leading to a set of 1,227 unique peptide candidates (Table S4). Cleavage sites had to be conserved in the MSA by at least 50% among all 74 organisms. For each candidate peptide, we extracted the peptide and precursor length and various evolutionary conservation scores based on rate4site calculations. Seven features were selected for a random forest classifier including: peptide length, precursor length, peptide mean, sum and standard deviation rate4site score and the mean score of the highest conserved stretch of 20 amino acids in the precursor and its ratio to the peptide mean score. A 5-fold cross validated model was trained on known human GPCR peptide ligands. For each run, a random split of 75% to 25% into training and test set was performed. The peptide classifier was based on the Random Forest algorithm (Breiman, 2001) implemented in Python *scikit-learn*. Peptides with a mean score of ≥ 0.45 were selected. The mean score (30%) and precursor length (27%) contributed most to the peptide selection. Peptide sequences with 1 or more than 2 cysteines were removed to prevent dimerization. The 131 predicted candidates were further clustered in CD-HIT to filter out similar peptides (> 0.85 sequence identity cut-off) (Huang et al., 2010). In total 120 peptide sequences were selected for synthesis from the machine learning approach. These peptides, together with a selection of 55 known class A peptide ligands and 43 (1 could not be synthesized) recently proposed peptides from mass spectrometry-based peptidomics on rat hypothalamus (Secher et al., 2016), formed a library of 218 peptide ligands for pharmacological assaying of orphan receptors (Table S4). The final library consisted of 49 known GPCR peptide ligands, 14 known peptide ligands with no previously shown GPCR activity, 35 variants of known peptides (i.e., containing parts of or overlap with known peptides) and 120 new peptides, which have not previously been described or associated with GPCR activity. Disulfide bridges were incorporated into 26 peptides containing two cysteines. 77 peptides containing an amidation consensus sequence motif (consisting of a glycine residue directly adjacent to the cleavage site after the peptide) were C-terminally amidated. No additional post-translational modifications such as bromination, glycosylation, sulfation and octanoylation were introduced.

#### Selection of peptide variants

Peptide variants were selected after visual inspection based on validated hits and alternative cleavage patterns with overlapping hit peptide regions. Variants had to be reasonably conserved in the precursor sequence evolutionary trace profile.

#### Generation of expression datasets

Gene expression for all 21 assayed orphan receptors was quantified through RNA sequencing (RNA-seq) data obtained from ARCHS4 RestAPI (Lachmann et al., 2018), which aggregates the majority of published RNA-seq data from human and mouse available at the gene and transcript levels with in total 187,946 samples. Human tissue expression levels were obtained for 53 distinct tissues grouped by system. Data were presented as median log_2._

#### Peptide library preparation and quality control

Peptides were purchased as a custom designed peptide library from Genscript, with > 95% purity cutoff and delivered as lyophilized acetate salts. In total, the library included 219 peptides varying in length from 3 – 50 amino acids, (full details and quality control data are found in Table S4). Peptides were resuspended to a stock concentration of 4 mM in 10% (v/v) DMSO/water and were diluted to 100 μM in water for in-house quality control. Peptides were analyzed by LC-MS (Agilent 6410 Triple Quadrupole LC/MS instrument using electron spray ionization (ESI) and coupled to an Agilent 1200 HPLC system) and UPLC (Waters Acquity H-class). One further peptide was excluded as it was insoluble in 10% (v/v) DMSO/water, leaving 218 peptides to assay.

Peptide stocks were diluted to 400 μM in Hank’s Balanced Salt Solution (HBSS) (GIBCO) containing 1% (v/v) DMSO and 0.01% (v/v) pluronic acid F68 (Thermo Fisher Scientific) and were plated duplicate wells of low-binding 384-well polypropylene daughter plates for storage at −20°C prior to screening.

#### Generation of orphan GPCR cell lines

##### Design and cloning of SNAP-tagged orphan GPCR constructs

pcDNA5/FRT/TO FLAG SNAP GPCR constructs were generated as described previously (Pedersen et al., 2019). In brief, an expression cassette containing the human interleukin 2 receptor alpha (hIL-2Ra) signal peptide, FLAG-tag and 19.3 kDa SNAP-tag in frame with unique 3′ MluI and 5′ NotI restriction sites for receptor insertion was introduced into the pcDNA5/FRT/TO vector (Thermo Fisher Scientific) using BamHI and NotI. The vector was modified by site-directed mutagenesis to remove an endogenous MluI site. Human orphan receptor coding sequences were modified to remove predicted signal peptides (Petersen et al., 2011), methionine start codons and any internal MluI and NotI restriction sites, and were purchased from Genscript. Orphan receptor sequences were subcloned into the MluI and NotI sites of the pcDNA5/FRT/TO FLAG SNAP vector. An expression construct expressing SNAP-tagged human glucagon-like peptide 1 (GLP-1) receptor was generated as a control cell-line for internalization assays (Foster and Bräuner-Osborne, 2018). All receptor constructs were confirmed by sequencing and can be found in Table S4.

##### Preparation of stable cell lines for orphan GPCR experiments

Low passage (less than P6) Flp-In T-REx 293 cells were grown to 80% confluency in 10 cm dishes in complete medium without zeocin and transfected using Lipofectamine 2000 (Thermo Fisher Scientific). Plasmids encoding SNAP-tagged oGPCR constructs were co-transfected with pOG44 Flp-Recombinase expression vector (Thermo Fisher Scientific) as per the manufacturer’s recommendations. After 24 hr, transfected cells were trypsinized and replated at low density into 10 cm dishes. 48 hr after transfection, stable transfectants were selected with 200 μg/mL hygromycin B. After ∼3 weeks of selection, stable cell lines were expanded to make stocks for storage at −150°C.

##### Cell-surface receptor expression using ELISA

To measure cell-surface receptor expression levels for oGPCRs during screening and functional assays, stable cell lines were plated at 30,000 cells/well in poly-D-lysine coated 96-well plates and induced with 0.1 μg/mL doxycycline for 48 hr at 37°C and 5% CO_2_ (Foster and Bräuner-Osborne, 2018). Immunodetection of amino-terminal FLAG epitope was performed as described previously (Jacobsen et al., 2013) with some modifications. Cells were fixed in 4% paraformaldehyde in PBS, washed and blocked, and then incubated with anti-FLAG M2 primary antibody (Sigma Aldrich) at room temperature for 45 min. HRP labeled anti-mouse secondary antibody (Vector Laboratories) was added for 45 min at room temperature and then cells were washed thoroughly prior to luminescence detection (SuperSignal ELISA Femto Substrate, Thermo Fisher Scientific). Detection solution was added (1:9 dilution in PBS) and chemiluminescence was measured immediately using an EnSpire multimode plate reader (PerkinElmer).

#### Functional assays to measure orphan GPCR activation

##### Peptide library screen

In order to assess the peptide-dependent orphan receptor responses, the peptides were tested in parallel DMR and real-time internalization assays. Peptide library daughter plates were thawed at room temperature and diluted to 40 μM (4x final for DMR assays) and 20 μM (2x final for internalization assays) in HBSS with 0.1% (v/v) DMSO and 0.01% (v/v) pluronic F68.

48 hr prior to parallel assaying, SNAP-tagged oGPCR cells were plated (10,000 cells/well) in fibronectin-coated 384-well Epic biosensor microplates (Corning) and poly-D-lysine coated white 384-well plates (for internalization assays). SNAP- GLP-1 receptor was plated alongside orphan receptor cells as an internal assay control for internalization assays. Receptor-expression was induced with 0.1 μg/ml doxycycline.

##### Dynamic mass redistribution assays

DMR assays provide a single integrated readout of ligand-dependent cellular response, utilizing an optical biosensor in specialized microplates to detect wavelength shifts of reflected light as it passes through the biosensor and the lower portion of the cell (∼150-200 nm). As a signal pathway agnostic approach, DMR assays are well-suited to the study of GPCRs (Fang et al., 2008, Schröder et al., 2011). The mass redistribution response (Δ picometer (pm)) likely reflects a number of intracellular events, including cytoskeletal rearrangement, protein trafficking and receptor internalization. Orphan receptor-expressing cells were gently washed twice with assay buffer (HBSS with 1 mM CaCl_2_, 1 mM MgCl_2_, 20 mM HEPES, 0.01% pluronic F-68, pH 7.4) containing 0.1% DMSO and cells were equilibrated for 90 min at 22°C in the EPIC Benchtop (BT) System (Corning) to establish a stable DMR signal. After 3-5 min baseline recordings, peptides (10 μM) were added in duplicate wells using an electronic 384-channel Viaflo pipette (Integra Biosciences, Switzerland) and DMR responses were measured for 60 min at 22°C. 100 nM norepinephrine was included as an internal assay control to assess the cellular DMR response via endogenously-expressed adrenoceptors in the TREx 293 cells. Real-time data were processed using the BT to column converter MS-Excel macro (Corning) and analyzed in GraphPad Prism 7.0. DMR recordings in peptide screening experiments were buffer and solvent corrected and the area under the curve (AUC) was quantified. For subsequent validation experiments, peptides were diluted in assay buffer containing 0.1% DMSO and DMR assays were performed as above. Concentration-response curves for DMR responses were generated using buffer and solvent-corrected AUC or the maximum value between 0-10 min where peptides elicited clear peak signals, as appropriate. Data represent mean ± standard error of the mean (SEM) from at least 3 biologically independent experiments.

##### Real-time internalization assay

A TR-FRET real-time internalization assay was used to examine peptide-dependent changes in orphan receptor localization as described previously (Foster and Bräuner-Osborne, 2018). Surface-expressed SNAP-tagged receptors were labeled with a cell-impermeable luminescent terbium cryptate (100 nM SNAP Lumi4-Tb; Cisbio, France) for 1 hr at 37°C. Cells were washed to remove excess terbium and incubated with fluorescein-O-acetic acid (100 μM; Sigma Aldrich) for 5 min at 37°C. Peptides were added as for DMR assays, and internalization responses were recorded for 60 min at 37°C using an EnVision 2104 Multimode plate reader (PerkinElmer). 100 nM GLP-1 was used as a positive control for SNAP-GLP1R internalization. Real-time internalization responses were calculated based on TR-FRET (excitation 340 nm; emission 520 nm and 615 nm), and were expressed as the ratio between donor (terbium) and acceptor (fluorescein) emission. For peptide screening analysis (both DMR and internalization assays), we calculated the area under the curve from the real-time traces. As the magnitude of the responses was variable between orphan receptor targets, particularly for the SNAP-tag based internalization assay where the signal is dependent on receptor surface expression, the AUC values were normalized to their respective plate averages and expressed as fold change. Validation experiments for receptor internalization were performed as above. Internalization data was processed in MS-Excel and area under the curve was used to generate concentration-response curves using GraphPad Prism 7.0. Data represent mean ± standard error of the mean (SEM) from at least 3 biologically independent experiments.

##### β-arrestin recruitment (Tango) assay

The peptide library was additionally screened against 120 GPCR targets in parallel, including all available orphan receptors and 27 known class A peptide receptors using the Tango assay platform (Kroeze et al., 2015). Briefly, HTLA cells (a HEK293 cell line stably expressing a tTA-dependent luciferase reporter and a β-arrestin2-TEV fusion gene) were plated 10,000 cells/well in poly-L-lysine–coated clear-bottom white 384-well plates in DMEM supplemented with 10% FBS, 100 U/ml penicillin and 100 μg/ml streptomycin, 2 μg/ml puromycin and 100 μg/ml hygromycin B. 24 hr later, cells were transfected with Tango GPCR constructs using the calcium phosphate method (Jordan et al., 1996). The next day, culture medium was changed to DMEM + 1% dFBS and 10 μM peptides were added for overnight stimulation. Bright-Glo solution (Promega) was incubated for 15–20 min at room temperature, and luminescence was counted using a Trilux luminescence counter (Wallac).

For subsequent peptide validation experiments using Tango assays, HTLA cells were transiently transfected in 10 cm dishes with the receptor of interest using polyethylenimine (PEI) (Longo et al., 2013). After 24 hr, 25,000 cells/well were plated in clear bottom, white-walled 384-well plates. Assays were performed as described above, with luminescence measured using an EnSpire 2300 Multimode plate reader (PerkinElmer). Luminescence counts were exported into Excel and were analyzed using GraphPad Prism 7.

##### β-arrestin recruitment (PathHunter®) assay

PathHunter eXpress Assays (DiscoverX) were performed in 384-well format according to the manufacturer’s recommendations. GPR1 cells were incubated with peptides for 90 min and luminescence measured using an EnSpire 2300 Multimode plate reader (Perkin*Elmer).*

##### IP_1_ and cAMP accumulation assays

Peptide-dependent second messenger responses were measured using commercially available HTRF-based IP_1_ and cAMP kits (Cisbio) as described previously (Nørskov-Lauritsen et al., 2014). Receptor expression was induced for 48 hr with 0.1 μg/ml doxycycline in 10 cm dishes. For IP_1_ measurements (to measure G_q/11_), 2,500 cells/well were mixed with ligands in triplicate wells in low volume white 384-well plates at 37°C for 30 min. For cAMP assays, 2,500 cells/well were added in triplicate wells in white 384-well plates in the absence or presence of 3 μM forskolin (to measure G_s_ and G_i/o_ activation respectively). 100 μM IBMX was included to prevent cAMP degradation during 30 min stimulation on a plate shaker at room temperature. For both assays, HTRF was measured using an EnVision 2104 Multimode plate reader (PerkinElmer). FRET ratios (665/615 nm) were interpolated to IP_1_/cAMP concentrations using a standard curve according to manufacturer’s recommendations.

##### Ca^2+^mobilization assays

Peptide-dependent Ca^2+^ mobilization responses were measured in HEK293 stable cell lines (HA-OGR1 (GPR68) and vector pcDNA) (Saxena et al., 2012) that were seeded in 96-well plates (1 × 10^5^ cells/well) and cultured at 37°C with 5% CO_2_ to near confluence in DMEM with 10% FBS (Corning). Six hours prior to assay, medium was changed to pH 8.0 or pH 7.4 HAM’s F12 Nutrient Mixture (GIBCO) without FBS and transferred to a 37°C incubator without CO_2_. One hour prior to stimulation, cells were incubated in pH 8.0 or pH 7.4 HBSS with Ca^2+^ and Mg^2+^ (GIBCO), supplemented with 15 mM HEPES, 2 μM Fluo-4 AM (Invitrogen) and 1.5 mM probenecid (organic anion transporter to prevent Fluo-4 efflux) as described previously (Pera et al., 2018). Indicated peptides were pipetted using an automated system and emission signal was recorded at 525 nm after excitation at 485 nm using Flex Station III (Molecular Devices). Ca^2+^ mobilization responses were determined by subtracting basal fluorescence from the net peak of agonist-induced fluorescence. Ionomycin (1 μM) was used to calculate the maximum Ca^2+^ signal to normalize peptide-mediated responses. No peptide-induced calcium mobilization response was observed in control (vector-expressing) cells.

##### GloSensor cAMP assays

GPR68-mediated cAMP signaling was measured using GloSensor cAMP assays as published previously (Huang et al., 2015, Yu et al., 2019) with modifications as indicated below. Calcium- and magnesium-free HBSS was supplemented with different organic buffer reagents for different pH solutions, 20 mM 2-(N-morpholino)ethanesulfonic acid (MES, Alfa Aesar) for pH 6.00 – 6.70, 20 mM 4-(2-hydroxyethyl)-1-piperazineethanesulfonic acid (HEPES, Fisher) for pH 6.80 – 8.20, and 20 mM (tris(hydroxymethyl)methylamino)propanesulfonic acid (TAPS) for pH 8.30 – 8.60. The phosphodiesterase (PDE) inhibitor Ro 20-1724 (10 μM final, Cayman Chemical) was included in pH buffers to eliminate potential effect of PDE inhibition. HEK293T cells were transfected with a modified polyethylenimine (PEI, Polysciences) transfection method (Longo et al., 2013). For a 15-cm dish of HEK293T cells with 25 mL growth medium, 8 μg receptor DNA and 8 μg GloSensor DNA (Promega) were mixed in 1 mL of Opti-MEM medium followed by addition of 80 μL PEI reagents. The mixture was added to cells after 20 min incubation at room temperature. Overnight transfected cells were plated in poly-L-lysine (Sigma) coated 384-well white clear bottom plates (Greiner Bio-One, #781098) using DMEM (Corning) + 1% dialyzed FBS (Omega Scientific) for at least 6 h (up to 24 h). Cells were removed of medium before receiving 20 μL/well of 3.5 mM luciferin (Goldbio) prepared in assay buffer (HBSS, 20 mM TAPS, pH 8.40, 10 μM Ro 20-1724) for 1 h at 37°C. Luciferin loading solutions were removed and premixed drug solutions at desired pH conditions were added to cells (25 μL/well). Luminescence was measured 25-30 min after drug application. Results were normalized (basal and maximum responses in the absence of modulator were set to be 0 and 100%) and pooled for analysis using GraphPad Prism.

To extract allosteric parameters, normalized results were fitted to the allosteric operational model (Ehlert, 2005, Kenakin, 2005, Price et al., 2005) with the following constraints: orthosteric agonist binding affinity (K_A_ for protons) was set as equal to the potency (which is pH 6.80) in the absence of modulator; basal was set to 0 after normalization; allosteric efficacy cooperativity (β) was set to 1 since modulators had little effect on proton efficacy; allosteric ligand efficacy τ_B_ was set to be 0 as they do not have agonist activity alone. All of the other parameters, including allosteric affinity cooperativity (α), allosteric modulator binding affinity K_B_, orthosteric agonist (protons) efficacy τ_A_, system maximal response, and slope factor n were all globally shared for all datasets for each modulator. Since the orthosteric agonist (protons in this case) is always present in the receptor compartment, the allosteric ligand binding affinity (K_B_), defined as the dissociation equilibrium constant of allosteric modulator in the absence of orthosteric agonist (i.e., H^+^), does not have corresponding biological meaning and cannot be determined in binding assays. We therefore used an allosteric index, log(αβ/K_B_) (Kenakin, 2017a), which contains both allosteric binding affinity and cooperativity, to quantify the overall allosteric activity.

### Quantification and Statistical Analysis

For all orphan receptor functional assays, results are presented as mean ± standard error of the mean (SEM) based on at least 3 biologically independent experiments. Curve fitting and analysis of statistical significance was performed using Prism (7.0, GraphPad). Statistical parameters are reported in figure legends where appropriate.

### Data and Code Availability

All data that support the findings of this study have been provided in Tables S1, S2, S3, S4, S5, S6, and S7. Scripts are available from the corresponding authors on request.

## Supplemental Information

Table S1. Human GPCR Ligands, Related to Figures 1, 2, S1, and S2

Table S2. Fingerprint Similarities and Jaccard Indices, Related to Figures 2 and S2

Table S3. Difference Matrix of Multiple Sequence Alignment between Peptide and Non-peptide Receptors for the 45 × 50 (ECL2) Region, Related to Figure 3

Table S4. Assay and Candidate Peptide Library, Related to Figures 4, 5, 6, 7, S5, S6, and S7

Table S5. Orphan Receptor Selection and Cell Line Controls, Related to Figures 4, 5, 6, 7, S4, S5, S6, and S7

Table S6. Peptide Screening Data, Related to Figure 4

Table S7. Peptide-Receptor Validation and Variant Data, Related to Figures 4, 5, S4, S5, and S7

Video Abstract
